# Evaluation of Peptide Nucleic Acid Encapsulation in Ferritin Nanocages for Gene Silencing Applications

**DOI:** 10.1021/acs.biomac.5c01489

**Published:** 2025-10-24

**Authors:** Andrea Patrizia Falanga, Maria Vittoria Farina, Gabriele Cianfoni, Lorenzo Barolo, Chiara Di Meo, Giulia Elizabeth Borsatti, Francesca Ghirga, Bruno Botta, Luca Pisano, Nicola Borbone, Stefano D’Errico, Alessio Paone, Giorgia Oliviero, Deborah Quaglio, Paola Baiocco

**Affiliations:** † Department of Pharmacy, 9307 University of Naples Federico II, Via Domenico Montesano 49, Naples 80131, Italy; ‡ Department of Biochemical Sciences “Alessandro Rossi Fanelli”, 9311 Sapienza University of Rome, P.le A. Moro 5, Rome 00185, Italy; § Department of Chemistry and Technology of Drugs, Sapienza-University of Rome, Rome 00185, Italy; ∥ Department of Physiology and Pharmacology, Sapienza University of Rome, Rome 00185, Italy; ⊥ Department of Molecular Medicine and Medical Biotechnology, University of Naples Federico II, Via Sergio Pansini 5, Naples 80131, Italy

## Abstract

Peptide nucleic acids (PNAs) feature a neutral peptide-like backbone, providing nuclease resistance and potential for precision medicine and diagnostics through specific DNA and RNA binding. However, their therapeutic use is hindered by poor solubility and cell permeability. In this study, we demonstrated that negatively charged PNAs can be readily loaded into the polycationic Humanized Archaeoglobus Ferritin, namely, PA3.2-HumAfFt bioconjugate system, following a divalent-cation-triggered oligomerization technique. The versatility of PNA chemistry enabled the production of synthetic nucleic acid homologues with varying lengths and charges, ranging from positive to negative. We evaluated the loading performance of HumAfFt with and without chemical modifications and investigated the release dynamics of PNAs under conditions simulating the intracellular environment. Our findings demonstrated the effective uptake, release, and biological activity of PNAs in cancer cells, notably silencing the GAPDH gene with good efficiency. This evaluation paves the way for optimizing PNA-based therapeutics and broadening their applications.

## Graphical abstract

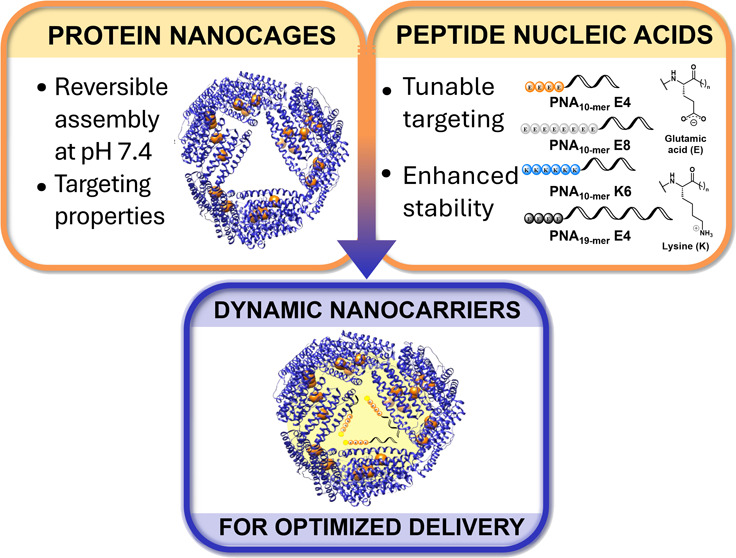

## 1 Introduction

Peptide nucleic acids (PNAs) are among the fascinating members of the xeno-nucleic acid family, featuring a neutral peptide-like backbone, i.e., *N*-(2-aminoethyl)­glycine.^1,2^ PNA oligomers can bind to complementary DNA or RNA sequences through Watson–Crick base pairing due to the nucleobases attached to their backbone. These features give PNAs superior properties, increasing their resistance to nucleases and proteases and improving their binding affinity and specificity, compared to natural oligonucleotides. Therefore, it is unsurprising that PNAs have gained significant interest in several research areas, from precision medicine therapeutics and chemical biology to molecular diagnostics.^3–5^ However, due to the neutral backbone, these nucleic acid analogues suffer from inherent weaknesses such as low aqueous solubility and cell permeability, which limit their use in many biomedical applications.^6^ Several strategies have been pursued to overcome these issues. Using standard solid-phase manual or automated synthetic techniques, conjugation with various modifiers, including peptides, has easily fine-tuned the PNA physicochemical properties.^7–9^ Taking a different approach, backbone-modified PNAs have been developed by incorporating specific side chains possessing unique chemical properties into the sequence of the PNA oligomer itself.^10^ Several multifunctional delivery systems have been developed for PNAs,^11–15^ i.e., liposome formulations,^16–19^ polymer nanoparticles,^20–24^ metal-based nanocarriers.^25,26^ While these strategies have shown promise, they also suffer from intrinsic limitations in terms of structural control, engineering flexibility, and overall biocompatibility. In contrast, protein containers provide a unique and innovative platform, offering well-defined structures, genetic manipulability, easy chemical modification, and superior biocompatibility.^27,28^ Specifically, ferritin (Ft) proteins, as naturally occurring, are used as smart nanocarriers for drug delivery due to their intrinsic nanocage architecture, which is made of 24 identical subunits. Favorably, this structure offers a confined internal space of 8 nm for molecular encapsulation while maintaining remarkable structural integrity.^29^ Many genetic modifications have attempted to enhance cargo loading properties of ferritin nanocages,^30,31^ which are cost-effective, thermally stable, and can be taken up by cancer cells through transferrin receptor 1 (TfR1 or CD71), which is overexpressed due to high iron demand.^29^ Recent structural findings highlight that the external unstructured loop region of human Ft is crucial for the TfR1 complex formation.^32^ A peculiar chimeric construct, namely, “Humanized Archaeoglobus Ferritin” (HumAfFt), emerged as an alternative to the human Ft homopolymers (Figure 1).^33,34^ Importantly, the external loop of HumAfFt incorporates a human-derived sequence, enabling recognition by the human TfR1 receptor through clathrin-mediated endocytosis for targeted delivery.^35^ This protein displays several advantages, including unusual salt-triggered assembly disassembly behavior at physiological pH, as well as the presence of a cysteine residue in each subunit, which allows for permanent chemical modification of the inner surface using tunable reagents. To counteract the intrinsically negative charge of ferritin, a versatile strategy has recently been implemented to increase the positive charge of its cavity, thereby promoting physical entrapment of oligonucleotides through chemical cross-linking of cyclic polyamines. Notably, pentafluorobenzene-based derivatives bearing an electron-withdrawing para substituent ensured thiol-selective modification of HumAfFt as maleimide-based reactive groups (Figure 1).^36,37^ This approach allows for rapid, mutation-free functionalization of the ferritin interior, enabling efficient and stable encapsulation of small RNAs featuring 22 nucleotides in length (i.e., siRNA and miRNA) without compromising the structural or biological properties of the protein shell.^38^

**Figure 1:**
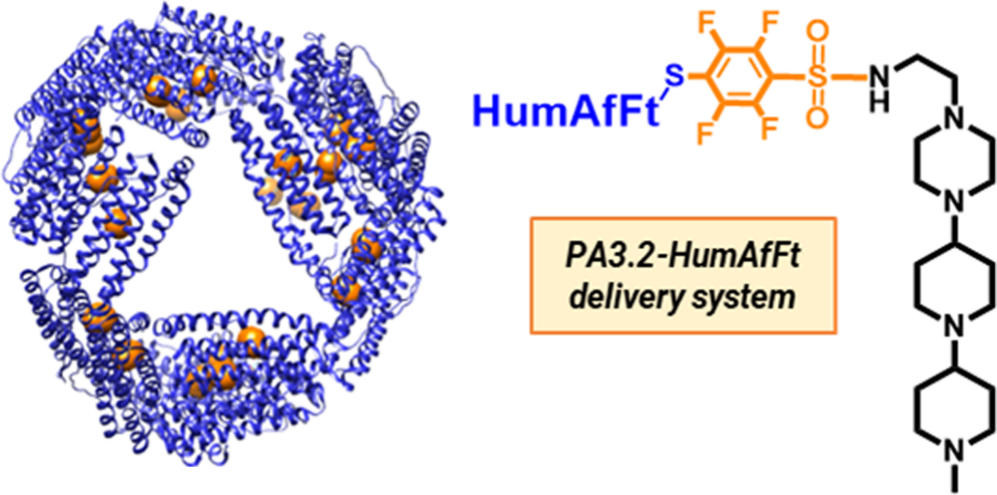
Schematic representation of the **PA3.2-HumAfFt** delivery system (orange spheres indicate the bioconjugation positions) (structural figures were prepared with UCSF Chimera).^39^

In the present study, we aimed to extend the charge-based encapsulation approach to load PNAs into the polycationic **PA3.2-HumAfFt** system using a divalent-cation-triggered oligomerization method. We provided synthetic PNAs with different lengths and net charges, ranging from positive to negative, and evaluated their loading performance with or without chemical modification in the HumAfFt protein cage. By exploring these variations, we aimed to identify the most effective conditions for encapsulation. Furthermore, we evaluated the stability of the PNA-loaded systems to gain insights into the dynamics of release and demonstrate the cellular uptake of the system, which is critical for developing an efficient delivery strategy for PNAs in potential biomedical applications.

## 2 Experimental Section

### 2.1 General Information

All reagents for the synthesis of PNA sequences were purchased from Merck KGaA and used without further purification. The solvents were dried and purified by standard laboratory methods. When not indicated, the reactions were carried out at room temperature (r.t.). High-performance liquid chromatography (HPLC) was performed using a Jasco UP-2075 Plus pump equipped with a Jasco UV-2075 Plus UV detector (Jasco Europe, Cremella, Italy) and a Macherey-Nagel 10 × 250 mm C-18 reverse-phase column with particle size of 5 μm (Macherey-Nagel, Düren, Germany) eluted with a linear gradient of CH_3_CN containing 0.1% trifluoroacetic acid (TFA) in H_2_O containing 0.1% TFA (from 0 to 100% CH_3_CN in 30 min, flow 3 mL/min). The amounts of each PNA were estimated spectrophotometrically using a Jasco V-530 spectrophotometer (λ = 220–450 nm, 400 nm/min scan speed, 2.0 nm bandwidth) using the molar extinction coefficients ε = 103,900 M^–1^ cm^–1^ for **PNA**_**10‑mer**_ **E4 (−)**, **PNA**_**10‑mer**_ **E8 (−)**, and **PNA**_**10‑mer**_ **K6 (+)**; ε = 117,600 M^–1^ cm^–1^for **FITC-PNA**_**10‑mer**_ **E4 (−)**, **FITC-PNA**_**10‑mer**_ **E8 (−)**, and **FITC-PNA**_**10‑mer**_ **K6 (+)**; ε = 208,500 M^–1^ cm^–1^ for **PNA**_**19‑mer**_ **E4 (−)**, and 222,200 M^–1^ cm^–1^ for **FITC-PNA**_**19‑mer**_ **E4 (−)**. The molar extinction coefficients were obtained using the “PNA tool” software at https://www.pnabio.com/support/PNA_Tool.htm. The molar extinction coefficients of FITC-labeled PNAs were obtained as described by Powell, Glen Research, in https://www.biosyn.com/faq/effect-on-quantifying-a-fluorescein-labeled-oligonucleotide-using-absorbance-at-260nm.aspx. The structures of pure PNAs were confirmed by electrospray mass spectrometry (ESI-MS) analyses performed on a 4000 QTRAP mass spectrometer (ThermoFisher Scientific, Waltham, MA, USA) in positive mode. All solvents and reagents for the synthesis of the PA3.2 linker were purchased from Merck KGaA (Darmstadt, Germany) or Carlo Erba Reagents (Milano, Italy) and used without further purification, unless otherwise stated. All reactions for the synthesis of the polyamine were monitored by thin-layer chromatography (TLC), performed on 0.2 mm-thick F254 silica gel plates. Flash column chromatography was performed by using neutral Al_2_O_3_ (Carlo Erba) as the stationary phase. ^1^H NMR spectra were recorded by using a Bruker 400 Ultra Shield spectrometer (operating at 400 MHz for ^1^H). The gene encoding a mutated ferritin from *Archaeoglobus fulgidus* was synthesized by GeneArt (ThermoFisher Scientific, Waltham, MA, USA) and subcloned into a pET22b vector (Novagen, Merck KGaA, Darmstadt, Germany) using the NdeI and *Hin*dIII restriction sites at the 5′ and 3′ ends, respectively. The recombinant plasmid was transformed into the BL21­(DE3) *E. coli* strain for protein expression (ThermoFisher Scientific, Waltham, MA, USA). All solvents and reagents for the purification of HumAfFt were purchased from Merck KGaA (Merck, Darmstadt, Germany). Protein samples were purified using an AKTA Pure system (Cytiva, Merck KGaA, Darmstadt, Germany).

### 2.2 PNA Synthesis and Analysis

The synthesis of all PNAs (Table 1) was performed using the 9-fluorenylmethoxycarbonyl (Fmoc) solid-phase synthetic protocol. After swelling 30 mg of Rink amide 4-methylbenzhydrylamine resin (loading: 0.5 mmol/g) in dimethylformamide (DMF) overnight, the first coupling with glycine was performed using the following conditions: 1.5 equiv of the Fmoc-Gly-OH monomer dissolved in DMF (0.2 M), 1.5 equiv of 1-[bis­(dimethylamino)­methylene]-1H-1,2,3-triazolo­[4,5-*b*]­pyridinium 3-oxide hexafluorophosphate (HATU) dissolved in DMF (0.2 M), and 2.25 equiv of *N*,*N*-diisopropylethylamine (DIPEA)/lutidine.

**Table 1:** Sequences of Synthesized PNAs Used in This Study

sample	sequence
**PNA**_**10‑mer**_ **E4 (−)**	H_2_N-(Glu)_4_-catgtaaacc-Gly-CONH_2_
**FITC-PNA**_**10‑mer**_ **E4 (−)**	FITC-(AEEA)_2_-(Glu)_4_-catgtaaacc-Gly-CONH_2_
**PNA**_**10‑mer**_ **E8 (−)**	H_2_N-(Glu)_8_-catgtaaacc-Gly-CONH_2_
**FITC-PNA**_**10‑mer**_ **E8 (−)**	FITC-(AEEA)_2_-(Glu)_8_-catgtaaacc-Gly-CONH_2_
**PNA**_**10‑mer**_ **K6 (+)**	H_2_N-(Lys)_6_-catgtaaacc-Gly-CONH_2_
**FITC-PNA**_**10‑mer**_ **K6 (+)**	FITC-(AEEA)_2_-(Lys)_6_-catgtaaacc-Gly-CONH_2_
**PNA**_**19‑mer**_ **E4 (−)**	H_2_N-(Glu)_4_-atattggaacatgtaaacc-Gly-CONH_2_
**FITC-PNA**_**19‑mer**_ **E4 (−)**	FITC-(AEEA)_2_-(Glu)_4_-atattggaacatgtaaacc-Gly-CONH_2_

Then, for assembling the PNA monomers, 3 equiv of each dissolved in DMF (0.2 M), 3 equiv of HATU in DMF (0.2 M), and 4.5 equiv of DIPEA/lutidine were used. After each coupling step, the capping step was performed in the presence of 20% acetic anhydride (Ac_2_O) and DIPEA in DMF for 10 min. Subsequently, the last Fmoc protecting group was removed through a double basic treatment (20% piperidine in DMF, 5 min each). After the syntheses of the 10-mer PNAs were completed, to allow the electrostatic interaction of PNAs with the cationic polyamines, four or eight residues of glutamic acid (E), bearing a negative side chain (COO^–^), were introduced at the *N*-terminus of the PNA sequences, namely, **PNA**_**10‑mer**_ **E4 (−)** and **PNA**_**10‑mer**_ **E8 (−)**, respectively, under the same conditions described before. Furthermore, a PNA bearing a six-lysine tail, namely, **PNA**_**10‑mer**_ **K6 (+)**, was synthesized to evaluate the loading capacity of the PNA in the cavity of HumAfFt without the positive linker.^40,41^ Based on the entrapment analysis, we selected the E4 tail as the optimal modification for the synthesis of the 19-mer PNA targeting GADPH, **PNA**_**19‑mer**_ **E4 (−)**.

For each PNA sequence, we also synthesized the corresponding fluorescein isothiocyanate (FITC)-labeled analogue, thus obtaining **FITC-PNA**_**10‑mer**_ **E4 (−)**, **FITC-PNA**_**10‑mer**_ **E8 (−)**, **FITC-PNA**_**10‑mer**_ **K6 (+)**, and **FITC-PNA**_**19‑mer**_ **E4 (−)**, as per the following protocol. Two coupling cycles with the 2-[2-(Fmoc-amino)­ethoxy]­ethoxyacetic acid linker (Fmoc-AEEA–OH) at the *N*-terminal groups of PNA were performed using the following conditions: 10 equiv of Fmoc-AEEA–OH dissolved in DMF (0.33 M), 10 equiv of HATU dissolved in DMF (0.33 M), and 10 equiv. DIPEA/lutidine. After the deprotection of the terminal amino groups, 5 equiv of FITC (0.2 M) were solubilized in 5 equiv. DMF/10 equiv of DIPEA, and the solution was added to the resin, which was gently stirred in the dark overnight. At the end of the syntheses, the crude PNAs were detached from the solid supports by treatment with trifluoroacetic acid (TFA)/anisole/ethanedithiol (9:1:1) for 2 h and precipitated with cold diethyl ether. After centrifugation, washing with diethyl ether (twice), and removal of solvents under nitrogen, the crude products were purified by HPLC. The fractions containing the pure compounds (overall yields of 87–99% for PNAs and 50–99% for FITC-labeled PNAs) were collected and lyophilized. The title compounds were characterized by ESI-MS (see Supporting Information, Figures S1–S16).

#### 2.2.1 PNA_10‑mer_ E4 (−)

ESI-MS (*m*/*z*) calcd. for [M + 2H]^2 +^ 1634.6, found 1635.0; calcd. for [M + 3H]^3 +^ 1090.1, found 1090.7; calcd. for [M + 4H]^4 +^ 817.8, found 818.6; calcd. for [M + 5H]^5 +^ 654.5; found, 655.1. **FITC-PNA**_**10‑mer**_ **E4 (−)**: ESI-MS (*m*/*z*) calcd. for [M + 2H]^2 +^ 1974.2, found 1974.6; calcd. for [M + 3H]^3 +^ 1317.5; found, 1317.4; calcd. for [M + 4H]^4 +^ 987.6; found, 988.4 calcd. for [M + 5H]^5 +^ 790.3; found, 791.0.

#### 2.2.2 PNA_10‑mer_ E8 (−)

ESI-MS (*m*/*z*) calcd. for [M + 3H]^3 +^ 1262.2; found, 1262.7 calcd. for [M + 4H]^4 +^ 946.9; found, 947.6 calcd. for [M + 5H]^5 +^ 757.7; found, 758.4 calcd. for [M + 6H]^6 +^ 631.6; found 632.1 calcd. for [M + 7H]^7 +^ 541.5; found, 541.9. **FITC**-**PNA**_**10‑mer**_ **E8 (−)**: ESI-MS (*m*/*z*) calcd. for [M + 3H]^3 +^ 1488.5; found, 1489.4; calcd. for [M + 4H]^4 +^ 1116.7; found, 1117.5 calcd. for [M + 5H]^5 +^ 893.5; found, 894.3 calcd. for [M + 6H]^6 +^ 744.8; found, 745.4 calcd. for [M + 7H]^7 +^ 638.5; found, 637.2.

#### 2.2.3 PNA_10‑mer_ K6 (+)

ESI-MS (*m*/*z*) calcd. for [M + 3H]^3 +^ 1174.2; found, 1175.0 calcd. for [M + 4H]^4 +^ 880.9; found, 881.6 calcd. for [M + 5H]^5 +^ 704.9; found, 705.5 calcd. for [M + 6H]^6 +^ 587.6; found, 588.1 calcd. for [M + 7H]^7 +^ 503.8; found, 504.3. **FITC-PNA**_**10‑mer**_ **K6 (+)**: ESI-MS (*m*/*z*) calcd. for [M + 3H]^3 +^ 1400.6; found, 1401.4; calcd. for [M + 4H]^4 +^ 1050.7; found, 1051.5 calcd. for [M + 5H]^5 +^ 840.8; found, 841.5 calcd. for [M + 6H]^6 +^ 700.8; found, 701.4 calcd. for [M + 7H]^7 +^ 600.8; found, 601.4 calcd. for [M + 8H]^8 +^ 525.9; found, 526.3.

#### 2.2.4 PNA_19‑mer_ E4 (−)

ESI-MS (*m*/*z*) calcd. for [M + 4H]^4 +^ 1438.1; found, 1439.3; calcd. for [M + 5H]^5 +^ 1050.7; found, 1051.5; calcd. for [M + 6H]^6 +^ 959.0; found, 960.2. **FITC-PNA**_**19‑mer**_ **E4 (−)**: ESI-MS (*m*/*z*) calcd. for [M + 4H]^4 +^ 1608.7; found, 1609.0; calcd. for [M + 5H]^5 +^ 1286.5; found, 1287.4; calcd. for [M + 6H]^6 +^ 1072.8; found, 1073.1; calcd. for [M + 7H]^7 +^ 919.7; found, 920.0.

### 2.3 “Humanized” Archaeoglobus Ferritin Expression and Purification

HumAfFt was designed by de Turris V. et al.^35^ with an M54C mutation per monomer to functionalize the protein inner cavity with sulfhydryl-reactive polyamines. *E. coli* cells containing the HumAfFt plasmid were cultivated and induced with 1 mM IPTG (isopropyl-β-D-1-thiogalactopyranoside) at OD600 = 0.6. After induction at 37 °C, cells were harvested by centrifugation after 3 h. The bacterial paste was then resuspended in a lysis buffer (300 mM NaCl and 20 mM HEPES pH 7.5, with two tablets of protease inhibitors, DNase, and 5 mM MgCl_2_), followed by sonication for 20 min and subsequent centrifugation for 30 min at 10000 rpm at 4 °C. The supernatant underwent a heating step at 78 °C with gentle agitation at 350 rpm for 10 min. Purification proceeded with two ammonium sulfate precipitation steps (at 40–70%), after which pellets were resuspended in 20 mM HEPES, pH 7.5, and 50 mM MgCl_2_, then dialyzed in 2 L of the same buffer solution. A gel filtration using a Sephacryl S400 column was performed as the final purification step. The purified protein was concentrated to yield a final preparation of 6 mg mL^–1^, with protein concentration determined using a spectrophotometer (Jasco V-750, Tokyo Instruments) by measuring the UV spectrum with an extinction coefficient of 32,400 M^–1^ cm^–1^. The protein yield was approximated to be 150 mg L^–1^ culture. The purified ferritin protein was sterilized by filtration using 0.22 μm filters and stored at 4 °C until needed.

### 2.4 Optimization of the PA3.2 Synthesis

The synthesis of the **PA3.2** linker is already reported in Pediconi et al.^36^ In the context of this study, we carried out an optimization of the synthetic procedures for preparing the final polyamine, increasing the total yield of the synthesis from 8 to 32% (see Supporting Information, Scheme S1). The main issues of the previously reported synthesis may be attributed to the challenging purification steps arising from the high polarity of the polyamines. In the reductive amination steps, the order of addition of the reagents was changed, introducing acetic acid into the reaction mixture before the reducing agent (NaBH­(OAc)_3_) to favor the formation of the intermediate iminium ion. All of the column purifications were carried out by using neutral Al_2_O_3_ as the stationary phase and a gradient mixture composed of chloroform (CHCl_3_), methanol (MeOH), and triethylamine (TEA) as the eluent phase (CHCl_3_ 100% → CHCl_3_/MeOH 99:1 + 1% TEA → CHCl_3_/MeOH 97:3 + 1% TEA). All the extractive workups were performed by using an aqueous NaOH solution (pH 14) to avoid the salification of the polyamines and dichloromethane (DCM) instead of CHCl_3_ as the organic solvent to prevent side reactions in a strongly basic environment. In particular, the workup of the hydrolysis reaction from **7** to **8** proved ineffective due to the high affinity of **8** for the aqueous medium, thus requiring the evaporation of the aqueous solution and sonication of the solid residue with DCM to drive the product to the organic solvent. Eventually, the purification of **PA3.2** by precipitation in hot hexane resulted in a partial solubilization of the target compound, leading to a partial product loss. Washings of the crude material with cold CHCl_3_—in which **PA3.2** is insoluble—led to crude residue purification and isolation of the final product in almost quantitative yield.

### 2.5 Preparation of the PA3.2-HumAfFt Bioconjugate

The purified HumAfFt was equilibrated in 20 mM HEPES, pH 7.5, and reduced by TCEP (Tris­(2-carboxyethyl)­phosphine hydrochloride) at a 10-fold excess per SH group for 1 h at 25°C under mild agitation. Simultaneously, **PA3.2** was solubilized in a 1:1 mixture of water and DMSO and introduced at a 10-fold excess relative to the targeted thiol group. The conjugation reaction took place overnight at 37 °C with gentle shaking at 300 rpm. After the reaction had reached completion, centrifugation removed any unreacted excess linker. A PD10 desalting column, pre-equilibrated with 20 mM HEPES at pH 7.5, was then used. Protein concentration was determined by using a spectrophotometer (Jasco V-750, Tokyo Instruments). The resulting sample was filtered through 0.22 μm filters for sterile preservation.

### 2.6 FITC-PNAs Encapsulation in HumAfFt

FITC-PNAs were resuspended in sterile, nuclease-free water at a final concentration of 1 mM (350 nmol in 350 μL) and stored at −20 °C. **PNA**_**10‑mer**_ **E4 (−)**, **PNA**_**19‑mer**_ **E4 (−)**, and **PNA**_**10‑mer**_ **E8 (−)** were encapsulated into the ***PA3.2-HumAfFt*** system, while **PNA**_**10‑mer**_ **K6 (+)** was encapsulated in HumAfFt with the desired excess fold compared to the HumAfFt (24-mer) structure. Under a biological safety hood, 3 μM HumAfFt in 20 mM HEPES, pH 7.5, was loaded with the desired excess of sterile PNAs. The mixture was gently agitated at 10 °C for 30 min. Finally, 100 mM MgCl_2_ was added (50 mM MgCl_2_ for **PNA**_**19‑mer**_ **E4 (−)**) and left overnight at 10 °C under gentle agitation. The HumAfFt–PNAs complexes were subsequently purified using PD25 mini columns and eluted with 1 mL of 20 mM HEPES, pH 7.5, and 50 mM MgCl_2_, with 500 μL of eluate collected. These complexes were then centrifuged for 2 min and 30 s at 14,000 rpm using concentrators with 50 kDa filters to remove unencapsulated PNA. The concentrated sample was 50 μL, so it was subsequently diluted with 350 μL of 20 mM HEPES, pH 7.5, and 50 mM MgCl_2_. Following this, UV–vis spectroscopy was utilized to measure the sample absorption at 280 and 494 nm, and the flow-through was obtained from the concentration steps. These peaks provided information about protein concentrations and the concentration of FITC-PNA. The FITC-PNA concentration was determined considering an extinction coefficient of 68,000 M^–1^ cm^–1^, and the protein concentration was determined by applying a correction factor to the absorbance at 280 nm to account for the contribution of FITC. The loading capacity was measured considering the ratio between FITC-PNA concentration over the protein concentration in 24 mer. The encapsulation efficiency (EE) was calculated using the following equation

$$EE\left(\%\right)=\frac{{\left[FITC-PNA-HumAfFt\right]}_{loaded}}{{\left[FITCPNA\right]}_{total}}\times 100$$

where FITC-PNA-HumAfFt loaded is the molar concentration of FITC-PNA experimentally determined in the protein complex over the molar concentration of FITC-PNA initially used to prepare the complex. Subsequently, DLS, ζ-potential, and Native-PAGE analyses were performed to characterize the physicochemical properties of plain HumAfFt and **PNA_10‑mer_E4 (−)-PA3.2-HumAfFt** complexes. DLS and ζ-potential measurements were conducted on samples prepared following the procedures described above for PA3.2 functionalization and FITC-PNA encapsulation at a 16-fold molar excess into HumAfFt. The sizes of HumAfFt and the PNA–PA3.2-HumAfFt complexes **PNA_10-mer_E4 (−)** and **PNA_19-mer_E4 (−)** were analyzed at 1 mg/mL in 20 mM HEPES and 50 mM MgCl_2_, by using a Zetasizer Nano ZS instrument (model ZEN3690, Malvern Instruments, Worcestershire, United Kingdom), equipped with a 5 mW HeNe laser (λ = 632.8 nm) at a scattering angle of 173°. ζ-potential measurements were performed on ***PA3.2-HumAfFt*** and **FITC-PNA_10‑mer_E4 (−)-PA3.2-HumAfFt** complexes at pH 7.5 and pH 5.0. For samples at pH 5.0, sodium acetate trihydrate (100 mM, pH 4.0) was added to the original buffer (20 mM HEPES, 50 mM MgCl_2_) to adjust the pH. Native-PAGE was conducted under nondenaturing conditions to assess the nanocage assembly state and qualitatively evaluate **FITC-PNA**_**10‑mer**_ **E4 (−)** encapsulation. Samples included free **FITC-PNA**_**10‑mer**_ **E4 (−)** at 50 μM, FITC-labeled HumAfFt at 1 mg/mL with an internal FITC concentration of 50 μM, **FITC-PNA_10‑mer_E4 (−)-PA3.2-HumAfFt** at 1 mg/mL prepared at pH 7.5 using a 16-fold molar excess of FITC-PNA, and the same **FITC-PNA_10‑mer_E4 (−)-PA3.2-HumAfFt** complex treated at pH 5.0 as described above. All samples were prepared in native loading buffer (100 mM Tris–HCl, 10% glycerol, pH 8.6), and 15 μL of each sample was loaded onto the gel alongside the Precision Plus Protein Standards (Bio-Rad Laboratories, San Francisco, USA) as a molecular weight marker. Electrophoresis was performed on a Mini-PROTEAN TGX Stain-Free gel (Bio-Rad Laboratories, San Francisco, USA) using Tris-Glycine Native running buffer (Invitrogen, Waltham, MA, USA) for 30 min at 200 V. Following electrophoresis, the gel was first imaged for FITC fluorescence using the Bio-Rad ChemiDoc touch imaging system (Bio-Rad Laboratories, San Francisco, USA) to visualize FITC-labeled species. Subsequently, the gel was stained with ProBlue Safe Stain (Giotto Biotech, Florence, Italy) to detect the total protein content and imaged again using the same system. The comparison between fluorescence and protein-stained images enabled qualitative evaluation of **FITC-PNA**_**10‑mer**_ **E4 (−)** encapsulation relative to ***PA3.2-HumAfFt*** content and assessment of the structural integrity of the nanocages under both pH conditions.

### 2.7 pH Dependency of FITC-PNAs on UV–Vis Analysis

FITC-PNAs were prepared and resuspended at 5 μM in 20 mM HEPES at pH 7.5 and 50 mM MgCl_2_. UV–vis spectroscopy, covering a wavelength range from 400 to 590 nm, was utilized to observe the absorbance at 494 nm, which provided information about the concentration of FITC-PNA (Jasco V-750, Tokyo Instruments). The concentration of FITC-PNA was determined using an extinction coefficient of 68,000 M^–1^ cm^–1^. Next, both samples were treated with 100 mM sodium acetate trihydrate at pH 4.0 to adjust the pH to 5.0, evaluating the influence of acidic pH on FITC quenching. The spectra of the samples were again obtained by using a UV–vis spectrophotometer as previously described. Finally, the samples adjusted to acidic pH were restored to neutral pH using 0.5 M HEPES at pH 7.5.

### 2.8 Release of FITC-PNAs from HumAfFt

***PA3.2-HumAfFt*** with 16-fold excess **PNA**_**10‑mer**_ **E4 (−)**, prepared as described previously, were subjected for 24 h of incubation in four different conditions: (1) control (CTR pH 7.5) at 4 °C, pH 7.5 in 20 mM HEPES, 50 mM MgCl_2_; (2) incubation at 37 °C, pH 7.5 in 20 mM HEPES, 50 mM MgCl_2_; (3) control (CTR pH 5.0) at 4 °C, pH 5.0 in 20 mM HEPES, 50 mM MgCl_2_, 100 mM sodium acetate trihydrate at pH 4; (4) incubation at 37 °C, pH 5.0 in 20 mM HEPES, 50 mM MgCl_2_, 100 mM sodium acetate trihydrate at pH 4. Following incubation, the samples were centrifuged for 2 min and 30 s at 14,000 rpm using concentrators with 50 kDa filters to aid in the removal of the released PNA. The supernatant and flow-through obtained were resuspended in 250 μL of 20 mM HEPES pH 7.5, 50 mM MgCl_2_, and analyzed using a NanoDrop 2000 spectrophotometer (Thermo Scientific, Waltham, MA, USA), covering a wavelength range from 240 to 600 nm. The protein concentration in monomers in the supernatant was determined considering an extinction coefficient of 32,400 M^–1^ cm^–1^, while the FITC-PNA concentration in the supernatant and flow-through was determined considering an extinction coefficient of 68,000 M^–1^ cm^–1^.

### 2.9 Cellular Uptake of FITC-PNA-HumAfFt

The MEG-01 cell line was obtained from ATCC (Manassas, VA, USA). Cells were maintained in RPMI-1640 medium (R8758, Sigma-Aldrich), supplemented with 10% fetal bovine serum (F7524, Sigma-Aldrich) and 100 IU/mL penicillin–streptomycin (P4458, Sigma-Aldrich). Cultures were incubated at 37 °C in a humidified atmosphere with 5% CO_2_. To evaluate the cellular uptake of **FITC-PNA**_**10‑mer**_ **E4­(−)** upon encapsulation in ***PA3.2-HumAfFt*** nanocages, three samples were prepared: free **FITC-PNA**_**10‑mer**_ **E4­(−)**, FITC-labeled HumAfFt, and **FITC-PNA_10‑mer_E4­(−)-PA3.2-HumAfFt**. Sample preparation followed the procedures described above for FITC-PNA resuspension, PA3.2 functionalization, and **FITC-PNA** encapsulation into ***PA3.2-HumAfFt*** with a 16-fold molar excess. All samples were sterile-filtered (0.22 μm) prior to cell treatment. Cells were seeded at a concentration of 180,000 cells per well in 12-well plates and incubated with a final concentration of 120 nM ***PA3.2-HumAfFt*** and 240 nM **FITC-PNA**_**10‑mer**_ **E4 (−)**, corresponding to the amount of PNA encapsulated under the applied loading conditions. After 6 h of treatment, suspension cells were collected into tubes, while adherent cells were gently detached using a cell scraper and collected into the corresponding tubes. All cells were then centrifuged for 5 min at 78 RCF, the supernatant was discarded, and the cell pellet was resuspended in PBS. Samples were kept on ice until fluorescence was measured using a NovoCyte 1040 flow cytometer (Agilent Technologies, Santa Clara, CA, USA).

### 2.10 RNA Extraction and Analysis

To evaluate the efficiency of gene silencing mediated by PNAs, cells were seeded at a concentration of 300,000 cells per well in 6-well plates and treated as previously described. After 48 h of treatment, cells were harvested and centrifuged at 78 RCF for 5 min, washed with PBS, and centrifuged again under the same conditions. Total RNA was extracted using the Quick-RNA Miniprep Kit (Zymo Research, Irvine, CA, USA), following the manufacturer’s instructions. One microgram of RNA was reverse-transcribed into complementary DNA using the high-capacity cDNA Reverse Transcription Kit (Cat. No. 4368814, Thermo Fisher Scientific). Quantitative PCRs were prepared in a final volume of 10 μL, consisting of 5 μL of SensiFAST SYBR Lo-ROX Kit (BIO-94005, Meridian Bioscience, Villa Cortese, Italy), 0.4 μL of forward primer (10 μM), 0.4 μL of reverse primer (10 μM), 1 μL of diluted cDNA (1:10), and 3.2 μL of nuclease-free water (129115, Qiagen). Amplifications were carried out using the QuantStudio 3 real-time PCR System (Applied Biosystems, Thermo Fisher Scientific) with the following cycling conditions: 50 °C for 2 min, 95 °C for 2 min, followed by 40 cycles of 95 °C for 5 s, and 60 °C for 30 s. Fluorescence was detected at the end of each cycle. A melting curve analysis was performed by using the default settings of the instrument to verify amplification specificity. Relative gene expression was calculated by using the 2̂−ΔΔ*Ct* method. Δ*Ct* values were determined by subtracting the *Ct* value of the housekeeping gene (β-actin) from the *Ct* value of the target gene. Each sample was analyzed in triplicate. Primer sequences are indicated in Section 6 of the Supporting Information.

## 3 Results and Discussion

### 3.1 Design and Synthesis of Chemically Diverse PNA Payloads

Recent investigations carried out in our laboratories revealed that the ***PA3.2-HumAfFt*** bioconjugate serves as an effective system for the encapsulation and subsequent release of small RNAs ranging from 18 to 25 nucleotides in length (Figure 1). This bioconjugate operates efficiently at a 1:1 ratio with the internal cavity of the protein, which allows for the binding and delivery of the RNA molecules. In the present study, to develop PNA molecules that can be hosted in the inner core of a chemically modified HumAfFt protein, we designed negatively charged PNA payloads targeting the 5′-GGUUUACAUGUUCCAAUAUU-3′ 20-mer RNA sequence located in the mRNA of the glycolytic enzyme glyceraldehyde 3-phosphate dehydrogenase (GADPH). PNAs are structurally designed to mimic DNA or RNA, allowing mixed-sequence PNA strands to hybridize with complementary single-stranded DNA or RNA. This results in the formation of highly stable heteroduplexes that exhibit exceptional sequence specificity. At moderate salt concentrations, the thermal stability of these duplexes increases, primarily because PNAs lack the negatively charged phosphate backbone found in DNA, which reduces interstrand electrostatic repulsion.^42–44^ Thus, considering the higher thermal stability of the PNA/RNA heteroduplex complex compared to the natural counterpart, we started our investigation by selecting a 10-nucleobase-long segment of the siRNA_GADPH_ sequence (5′-AUAUUGGAACAUGUAAACC-3′) previously reported in Pediconi *et al.* ^36^ The choice to employ a shorter PNA is founded on the effective application of 10-mer PNAs in antisense technology, specifically through the mechanism of translational arrest.^45^ For this purpose, we selected a pyrimidine-rich tract, given the enhanced yields associated with PNA coupling reactions. Using the OligoAnalyzer Tool (https://eu.idtdna.com), we verified that the PNA strand was not prone to fold into ordered secondary structures or self-dimers. Since physical entrapment into the ferritin nanocage is driven by electrostatic interactions between the cationic piperazine-based linkers and the uncharged PNA molecules, we inserted either four or eight glutamic acid residues at the *N*-terminus of the PNA sequences, which are negatively charged at physiological pH (p*K*_a_ = 4.25), thus obtaining **PNA**_**10‑mer**_ **E4 (−)** and **PNA**_**10‑mer**_ **E8 (−)**, respectively (Figure 2). To evaluate the ability of plain ferritin protein to load PNA without using polyamine linkers, we synthesized a positively charged PNA analogue, namely, **PNA**_**10‑mer**_ **K6 (+)**, by replacing the glutamate tail with a positively charged tail consisting of six lysine residues (Figure 2). Otherwise, neutral PNA was not employed in this study due to its complete lack of solubility, which results from the absence of electrostatic charge arising from the missing phosphate groups in its backbone, unlike natural DNA and RNA. Thus, after evaluating the loading efficiency of the PNAs containing the E4, E8, or K6 peptidyl chains, we aimed to define the best compromise between HumAfFt entrapment capacity, synthetic cost-effectiveness, and time efficiency. Consequently, we produced the full-length 19-mer PNA analogues of siRNA_GADPH_ featuring the E4 tail at their *N*-terminus (**PNA**_**19‑mer**_ **E4 (−)**, Table 1 and Figure 2).

**Figure 2:**
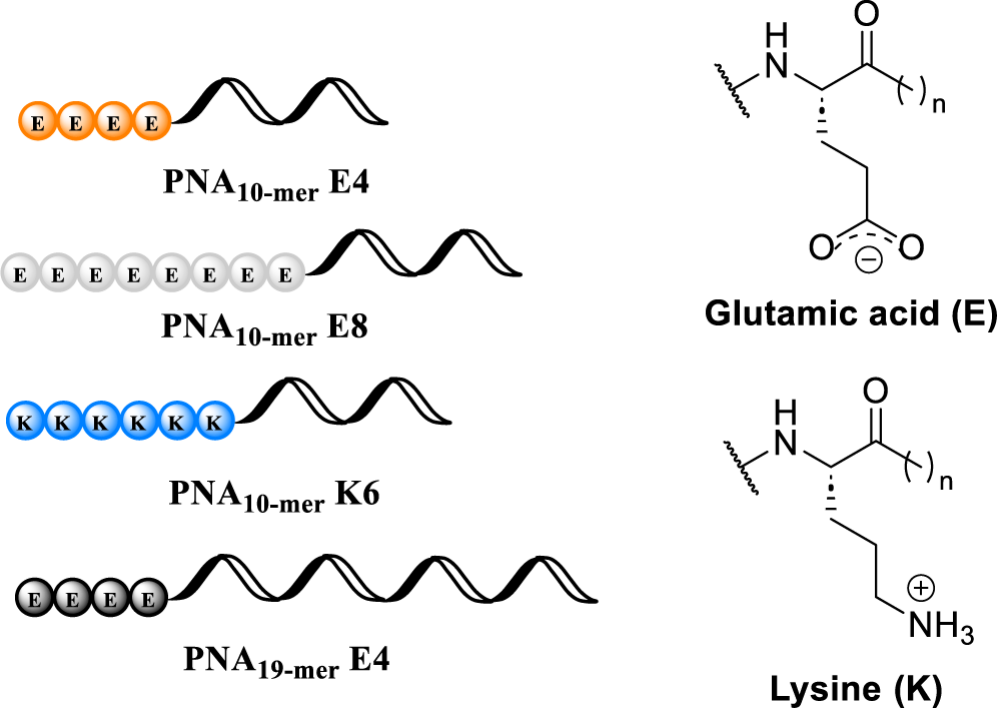
Schematic representation of PNA oligomers conjugated to differently charged amino acid residues.

All the PNA payloads were labeled with FITC to define the loading capacity and the release kinetics of PNA–PA3.2-HumAfF systems.

### 3.2 Characterization of the FITC PNA-HumAfFt Complexes

Our research focused on the encapsulation of the negatively charged PNAs within the ***PA3.2-HumAfFt*** system while also investigating the feasibility of encapsulating positively charged PNAs into the unmodified HumAfFt structure to evaluate the critical function of cationic linker **PA3.2**. To achieve this aim, we encapsulated increasing concentrations of FITC-labeled PNAs, relative to the protein concentration in 24-mer (ranging from 4 to 32-fold excess), into HumAfFt exploiting a disassembly/reassembly mechanism by the addition of MgCl_2_, as described in Figure 3.^35^ The maximum loading capacity for each construct, expressed as the number of PNA molecules per ferritin nanocage, was determined by measuring the ratio of the concentration of FITC dye to the concentration of 24-meric ferritin. The concentration of FITC was calculated by measuring the absorbance at 494 nm (ε = 68,000 M^–1^ cm^–1^).

**Figure 3:**
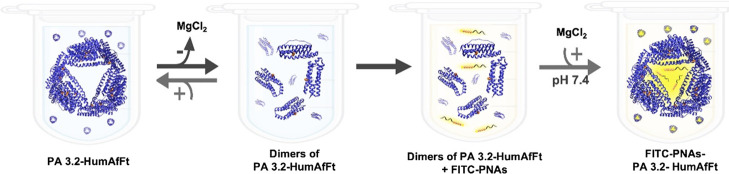
Schematic drawing of the encapsulation process of 10-mer FITC-PNA. The FITC-PNAs were added to the open conformation, and physical entrapment followed the closure of the nanocage by increasing salt concentration.

The protein concentration was calculated by measuring the absorbance at 280 nm, subtracting the contribution of FITC. As expected, increasing the FITC-labeled PNA/ferritin ratio led to an increase in the number of encapsulated molecules for each payload (Figure 4A). At lower ratios (4:1), the loading capacity appeared independent of PNA charge, likely due to statistical encapsulation within the nanoparticle. However, at higher ratios, negatively charged PNAs demonstrated superior loading capacity compared to positively charged PNAs. Specifically, in the case of **PNA**_**10‑mer**_ **E8 (−)**, saturation occurred at an average of five PNA molecules per ferritin (Figure 4A, gray). A similar pattern was observed for **PNA**_**10‑mer**_ **E4 (−)**, which reached saturation at four molecules per ferritin (Figure 4A, orange). In contrast, **PNA**_**10‑mer**_ **K6 (+)**, a positively charged PNA, exhibited consistently low incorporation levels into unmodified HumAfFt, even when used in excess (Figure 4A, blue). These results demonstrate that charge-based interactions play a crucial role in the encapsulation of artificial oligonucleotide analogues and highlight the significant advantages of a chemically modified protein cavity in enhancing the encapsulation efficiency.

**Figure 4:**
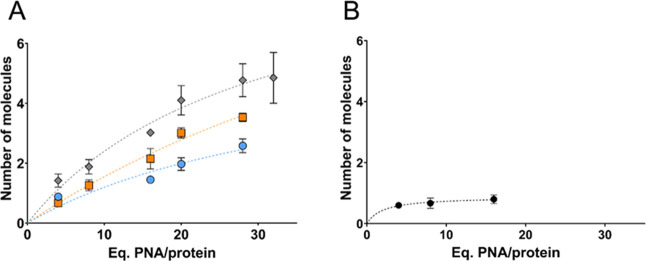
Encapsulation of 10-mer and 19-mer PNAs with the desired excess fold compared to the HumAfFt (24-mer) structure. The *x*-axis represents different complexes of HumAfFt with their respective excesses of PNA. The *y*-axis indicates the number of PNA molecules encapsulated within HumAfFt relative to the treated excesses. Measurements are conducted in triplicate. (A) The loading capacity of **PNA**_**10‑mer**_ in ***PA3.2-HumAfFt*** is shown. In blue, **PNA**_**10‑mer**_ **K6 (+)**; in orange, **PNA**_**10‑mer**_ **E4 (−)**; in gray, **PNA**_**10‑mer**_ **E8 (−)**. (B) The loading capacity of **PNA**_**19‑mer**_ **E4 (−)** in ***PA3.2-HumAfFt*** is shown. Error bars are lower than 4%.

Then, to evaluate the impact of longer structures on the encapsulation process in ***PA3.2-HumAfFt***, the loading capacity for **PNA**_**19‑mer**_ **E4 (−)** was calculated. The results demonstrated that the maximum loading capacity is limited to 0.8 molecules of PNA per HumAfFt, irrespective of the excess PNA used. This suggests that ferritin is unable to encapsulate these larger and more rigid structures beyond this capacity (Figure 4B).

### 3.3 Encapsulation Efficiency of FITC–PNAs into (Un)­modified HumAfFt

The encapsulation efficiency (EE) of PNAs into HumAfFt was assessed by using the equation in Section 2.6. The results revealed that the encapsulation efficiency varied among different types of PNAs. In particular, **PNA**_**10‑mer**_ **E8 (−)** demonstrated a notably higher encapsulation efficiency of 20.3 ± 2.0%. This was significantly greater than that observed for **PNA**_**10‑mer**_ **E4 (−)**, which had an efficiency of 13.7 ± 1.0%, as well as **PNA**_**19‑mer**_ **E4 (−)** and **PNA**_**10‑mer**_ **K6 (+)**, both of which exhibited considerably lower efficiencies of 9.4 ± 4.2% and 9.4 ± 0.3%, respectively. These findings elucidate that the interaction between the negatively charged PNAs and the positively charged polycationic linker is crucial and effective, leading to better results compared to an unmodified protein cage. Moreover, the study noted a trend, indicating that larger PNAs are less efficiently encapsulated than their smaller counterparts. This reduced efficiency is likely influenced by the internal dimensions of the encapsulating cavity, which is 8 nm. The rigidity introduced by the incorporation of peptide bonds within the complex three-dimensional structure of these PNAs may also play a significant role in limiting their encapsulation capacity. The results from dynamic light scattering (DLS) analysis provided valuable insights into the characteristics of the HumAfFt protein after PNA encapsulation (see Supporting Information, Figure S18). It was found that HumAfFt has a mean particle size of 14.3 ± 0.6 nm, indicating a relatively narrow distribution with a polydispersity index (PDI) of 0.13, suggesting uniformity in size. The **PA3.2-HumAfFt**/**PNA**_**10‑mer**_ **E4 (−)** complex demonstrated a 93.5% population with a slightly higher mean size of 15.2 ± 0.1 nm, accompanied by an even lower PDI of 0.2. Additionally, measurement of the ***PA3.2-HumAfFt***/**PNA**_**19‑mer**_ **E4 (−)** complex revealed a 99.2% population with a mean size of 15.4 ± 0.6 nm, maintaining a comparable PDI of 0.2. This consistent sizing across the various complex formations indicates stable interactions within the complex.

To further investigate the physicochemical properties of the biomolecular nanocage, ζ-potential measurements were carried out (see Supporting Information, Figure S19). The ***PA3.2-HumAfFt*** nanocage exhibited a surface potential of −9.78 ± 0.46 mV at physiological pH. After encapsulation with **PNA**_**10‑mer**_ **E4 (−),** the **ζ**-potential slightly increased to −4.75 ± 0.29 mV at pH 7.5 and to −5.24 ± 0.37 mV at pH 5.0. These shifts indicate an interaction between the nanocages and **PNA**_**10‑mer**_ **E4­(−)**. Native-PAGE analysis was subsequently employed under nondenaturing conditions to probe the structural integrity of ***PA3.2-HumAfFt*** nanocages and assess the encapsulation of FITC-labeled **PNA_10‑mer_E4­(−)** (see Supporting Information, Figure S20). Fluorescence imaging (see Supporting Information, Figure S20A) showed that free **FITC-PNA**_**10‑mer**_ **E4­(−)** migrated quickly through the gel, consistent with its small molecular weight. In contrast, FITC-labeled HumAfFt displayed a prominent fluorescent band at a higher molecular weight, matching the expected size of the assembled ferritin nanocage. Samples containing **FITC-PNA_10‑mer_E4­(−)-PA3.2-HumAfFt** complexes at both pH 7.5 and 5.0 also exhibited fluorescent bands comigrating with the FITC-HumAfFt control. This indicates that the FITC signal is associated with a high-molecular-weight species, suggesting encapsulation of the PNA within the nanocages. To confirm this, the same gel was subsequently stained with Coomassie (see Supporting Information, Figure S20B), revealing protein bands at the same positions as the fluorescent signals. The overlap of fluorescent and Coomassie-stained bands supports that the FITC-labeled PNA is indeed colocalized with the protein cages, verifying its association with the nanocage complexes. Additionally, a slight upward shift in the electrophoretic mobility of the **FITC-PNA_10‑mer_E4­(−)-PA3.2-HumAfFt** complexes, compared to the unmodified FITC-HumAfFt, further supports the presence of both the **PA3.2** functionalization and internalized PNA. Taken together, these data demonstrate that the ***PA3.2-HumAfFt*** nanocages retain their native structure after functionalization and effectively encapsulate the PNA molecules.

### 3.4 Release of Encapsulated FITC-PNAs from Modified-HumAfFt

Based on the encapsulation results of the negatively charged 10-mer PNAs in HumAfFt, we focused on examining the release capacity of encapsulated FITC-PNAs. Our investigation particularly considered how temperature and pH conditions affect the release process after 24 h. To mimic physiological conditions, we started our analysis considering the release at 37 °C compared to 4 °C and pH 7.5 compared to the acidic pH typical of a lysosomal environment through the measurement of free FITC-PNA and compared to the initial concentration calculated from the absorption of FITC at 494 nm. A wide variety of methods have been explored to determine the drug release from nanocarriers including dialysis and centrifugation techniques through 50 kDa units. Importantly, the release profiles obtained under physiologically relevant conditions are strongly affected by a well-documented quenching phenomenon related to the FITC sensitivity to acidic pH.^46,47^ To address this challenge, we decided to analyze the stability of **FITC-PNA**_**10‑mer**_ under various conditions. We prepared a control (CTR) consisting of FITC-PNA at pH 7.5. Subsequently, we prepared samples at pH 5.0 and then re-equilibrated the pH to neutral. Through these analyses, we observed a shift and splitting of the peak usually observed at 494 nm, moving toward shorter wavelengths, resulting in a decrease in the absorbance value compared to the control. Furthermore, upon adjusting the pH of the samples from 5.0 to 7.5 to assess if improvements in the peak were observed, we found that the splitting was resolved and the usual peak of FITC at 494 nm was restored, but with lower absorbance values (Figure 5). The data highlights that samples analyzed at pH 5.0 cannot be reliably normalized with samples not treated under the same conditions. Therefore, we decided to include a pH 5.0 control in our release analysis to reduce the variability caused by this condition.

**Figure 5:**
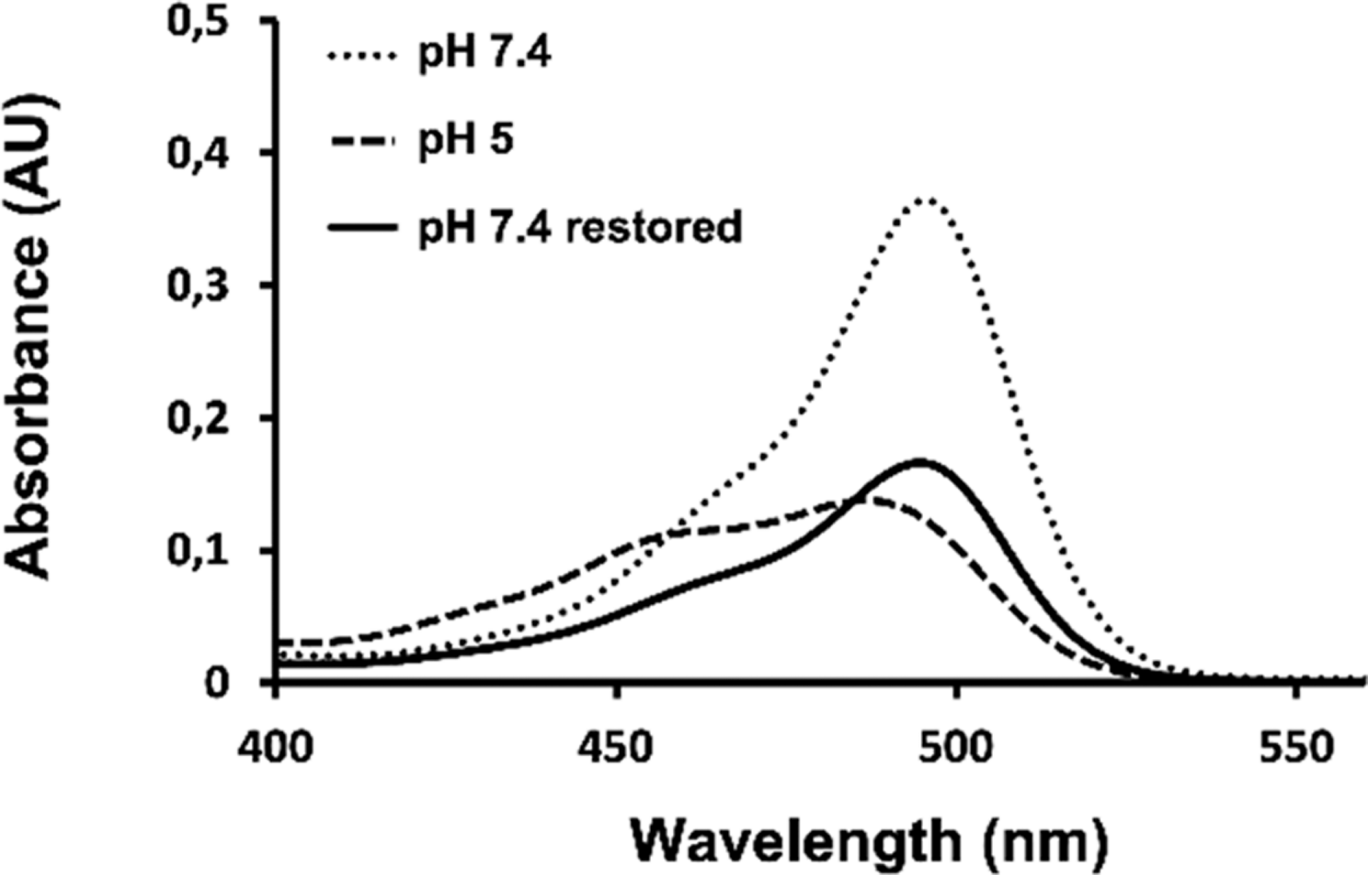
Spectra of FITC-PNA behavior under various pH conditions. Spectra of **FITC-PNA**_**10‑mer**_ 5 μM at pH 7.4 (dots), **FITC-PNA**_**10‑mer**_ at pH 5.0 (dashed line), and **FITC-PNA**_**10‑mer**_ adjusted from 5.0 to 7.4 (black line) showed the decrease of absorption intensity at 494 nm with the lowering of pH.

Once the reliability of our measurements was assessed, the release of encapsulated FITC-PNAs into ferritin nanoparticles was evaluated in tube assays as a function of temperature and pH to simulate cellular conditions.^48,49^ Accordingly, the release of **FITC-PNA**_**10‑mer**_ cargos was quantified at 37 °C and pH 7.4 and 5.0 using 50 kDa concentrator filters, separating HumAfFt from the released **FITC-PNA**_**10‑mer**_ payload collected in the filtrate. Concentrations of released **FITC-PNA**_**10‑mer**_ were measured based on absorbance at 494 nm (ε = 68,000 M^–1^ cm^–1^). We prepared four samples by encapsulating a 16-fold excess of **FITC-PNA**_**10‑mer**_ **E4 (−)** into modified HumAfFt and subjected them to different incubation conditions varying in temperature and pH. Under control conditions (CTR, pH 7.5 and pH 5.0), the basal release of **FITC-PNA**_**10‑mer**_ **E4 (−)** was observed, and the release efficiencies were calculated as the percentage of released **FITC-PNA**_**10‑mer**_ **E4 (−)** relative to the initial encapsulated amount, reaching 50%. The release efficiency increased to 70.5 ± 2% at 37 °C and pH 7.5 and was further enhanced to 81.5 ± 6% at 37 °C under acidic conditions (pH 5.0; Table 2).

**Table 2:** Loading Capacity, Encapsulation Efficiency (E.E.), and Release Efficiency (R.E.) of FITC-Labeled PNAs in ***PA3.2-HumAfFt*** Nanocarriers^a^

sample	loading capacity	E.E (%)	R.E. 37 °C (%)	R.E. 37 °C pH 5.0 (%)
**FITC-PNA**_**10‑mer**_ **E4 (−)**	4 ± 0.14	13.7 ± 1.0	70.5 ± 2.0	81.5 ± 6.0
**FITC-PNA**_**10‑mer**_ **E8 (−)**	5 ± 0.54	20.3 ± 2.0	n.d.	n.d.
**FITC-PNA**_**19‑mer**_ **E4 (−)**	0.8 ± 0.14	9.4 ± 4.2	n.d.	n.d.
**FITC-PNA**_**10‑mer**_ **K6 (+)**	1.8 ± 0.23	9.4 ± 0.3	n.d.	n.d.

a Loading capacity is expressed as the number of FITC-PNA molecules per nanoparticle. Since FITC fluorescence is pH-sensitive, for the release experiments, we included pH-matched FITC controls and report release values corrected for FITC quenching. All values are means ± SD of *n* = 3 independent replicates.

These results demonstrate that HumAfFt effectively encapsulates negatively charged **FITC-PNA**_**10‑mer**_ molecules and releases them in response to specific environmental conditions (Table 2). Both **PNA**_**10‑mer**_ **E8 (−)** and **E4 (−)** showed efficient loading into PA-HumAfFt. Due to its more reproducible encapsulation profile under our experimental conditions, **E4 (−)** was chosen for release studies and subsequent cellular uptake experiments. Since both PNA variants follow the same loading mechanism, the successful incorporation of **E4 (−)** provides strong evidence that **E8 (−)** would also be efficiently accommodated. The observed pH- and temperature-responsive release behavior highlights the potential of this system to operate in physiological and pathological environments such as mildly acidic tumor microenvironments, lysosomal compartments, and blood circulation. Each measurement was performed in triplicate, representing the average of three technical replicates. These results demonstrate that HumAfFt effectively encapsulates negatively charged **FITC-PNA**_**10‑mer**_ molecules and releases them in response to specific environmental conditions. This pH- and temperature-responsive release behavior may be particularly relevant in physiological and pathological environments such as mildly acidic tumor microenvironments, lysosomal compartments, and blood circulation.

### 3.5 Cellular Uptake of FITC- PNA_10‑mer_ E4 (−) Loaded-PA-HumAfFt

To assess the actual uptake of PNA molecules, we used fluorescent FITC-labeled PNAs and analyzed treated samples by flow cytometry. MEG01 cells, known to express high levels of CD71,^38^ were incubated under three different conditions: untreated, treated with free **FITC-PNA**_**10‑mer**_ **E4 (−)**, or treated with **FITC-PNA**_**10‑mer**_ **E4 (−)** encapsulated within ferritin nanocages. Six hours post-treatment, cells were collected, washed with PBS, and prepared for flow cytometric analysis. As shown in Figure 6, untreated cells (Ctr) served as a nonfluorescent control. Notably, cells treated with **FITC-PNA**_**10‑mer**_ **E4 (−)** exhibited fluorescence levels comparable to the control, indicating that the unencapsulated PNA was completely washed away and did not enter the cells, ruling out false-positive signals. In contrast, cells treated with **FITC-PNA**_**10‑mer**_ **E4 (−)**-loaded ***PA3.2-HumAfFt*** showed a clear fluorescent signal, demonstrating that ferritin nanocages are essential for cellular uptake.

**Figure 6:**
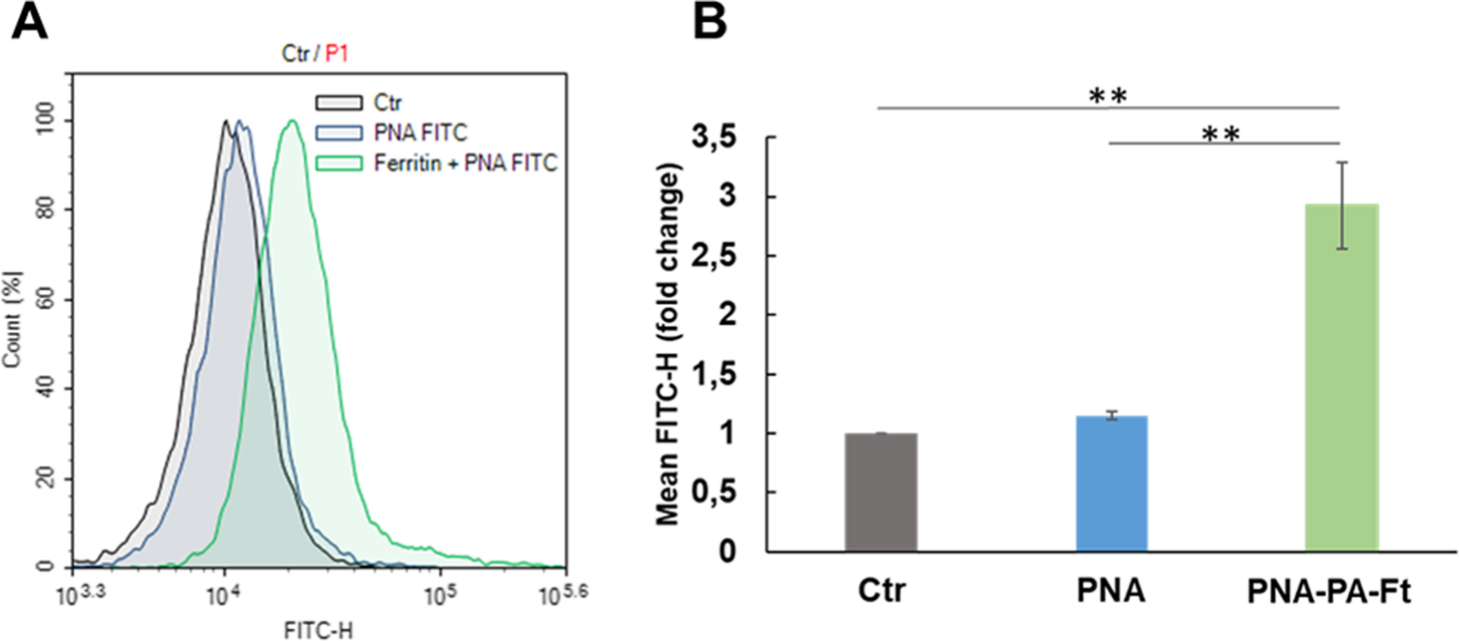
PNA-loaded ***PA3.2-HumAfFt*** uptake: (A) flow cytometry analysis of the negative control (black line), **FITC-PNA**_**10‑mer**_ **E4 (−)** alone (blue line), and **FITC-PNA**_**10‑mer**_ **E4 (−)** loaded into ***PA3.2-HumAfFt*** (green line) after 6 h of incubation in MEG-01 cells. Representative data of three independent experiments with similar results. (B) Mean fluorescence intensities of cellular uptake are depicted as histograms showing mean values ± SD from 3 independent measurements. ** = *p* < 0.001.

### 3.6 Targeting Efficiency of Ferritin-Encapsulated PNA_10‑mer_ E4 (−) against GAPDH

To assess the effectiveness of the targeting capability of **PNA**_**10‑mer**_ **E4 (−)** encapsulated within ferritin nanocages, we evaluated the expression levels of a previously characterized target, GAPDH.^36^ Cells were treated with three different conditions: (i) **PNA**_**10‑mer**_ **E4 (−)** alone (PNA), which, as demonstrated in Figure 6, is not capable of cellular uptake and thus served as a negative control; (ii) **PNA**_**10‑mer**_ **E4 (−)** encapsulated within ferritin nanocages PNA–PA-Ft); and (iii) a previously validated smart pool of four siRNAs targeting GAPDH (RNAi-TX) delivered via a standard transfection agent (jetPRIME, code 101000027, Polyplus, Life Sciences).^15,36^ As shown in Figure 7, treatment with ferritin-encapsulated **PNA**_**10‑mer**_ **E4 (−)** resulted in an approximately 45% reduction in GAPDH expression compared to treatment with **PNA**_**10‑mer**_ **E4 (−)** alone. Notably, although the silencing efficiency observed with the ferritin-encapsulated **PNA**_**10‑mer**_ **E4 (−)** appeared higher than that achieved using the standard siRNA transfected with a conventional reagent, the difference did not reach statistical significance.

**Figure 7:**
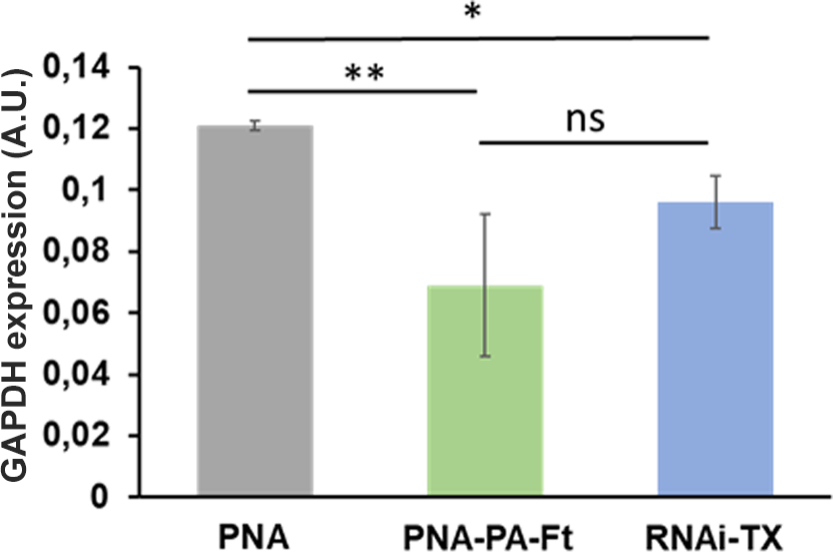
Real-time PCR analysis of GAPDH expression. Cells were treated with **PNA**_**10‑mer**_ **E4 (−)** alone (PNA), **PNA**_**10‑mer**_ **E4 (−)** loaded into ***PA3.2-HumAfFt*** nanocages (PNA–PA-Ft), or siRNA targeting GAPDH (RNAi-TX), and transfected with a jetPRIME transfection agent. Data represent the mean of three independent experiments. *p < 0.05; **p < 0.001; n.s., not significant. Statistical significance was assessed using one-way ANOVA, followed by the Bonferroni post hoc test.

## 4 Conclusions

In our study, we successfully demonstrated that negatively charged PNAs can be efficiently encapsulated within a polycationic protein cage known as ***PA3.2-HumAfFt***. This encapsulation process was achieved using a divalent-cation-triggered oligomerization technique that has been previously validated for the loading of negatively charged oligonucleotides. The unique and versatile chemistry of PNAs enabled the synthesis of RNA analogues with varying lengths and tailored net charges, allowing for customized design according to specific experimental needs. Specifically, we developed charged PNA payloads based on a 10-nucleobase segment of the siRNA_GADPH_ sequence. These PNAs were designed to include a pyrimidine-rich segment, facilitating the synthesis of a readily manageable material and exhibiting stability in mildly acidic biological environments. To further improve the interaction with the cationic linkers, we created **PNA**_**10‑mer**_ **E4 (−)** and **PNA**_**10‑mer**_ **E8 (−)**, both of which included additional glutamic acid residues. In contrast, a positively charged variant, **PNA**_**10‑mer**_ **K6 (+)**, was synthesized by substituting the glutamate with lysine residues to explore differences in loading efficiency. Interestingly, we observed that the loading efficiency was largely charge-independent at lower ratios of PNAs to the protein cage (specifically, a 4:1 ratio). However, at higher ratios, negatively charged PNAs exhibited better performance while preserving the natural size of the nanoparticle. Notably, **PNA**_**10‑mer**_ **E8 (−)** demonstrated saturation at five molecules per ferritin particle, whereas the larger **PNA**_**19‑mer**_ **E4 (−)** was constrained to only 0.8 molecules per HumAfFt particle. The encapsulation efficiencies of the various PNAs exhibited a significant variance. Among the tested sequences, **PNA**_**10‑mer**_ **E8 (−)** showcased the highest encapsulation efficiency at 20.3 ± 2.0%, while **PNA**_**10‑mer**_ **E4 (−)** achieved an acceptable 13.7 ± 1.0%. Concerning the release testing, our findings revealed that acidic conditions significantly influenced the release profiles. Under physiological conditions, specifically at 37 °C and a neutral pH of 7.5, the release efficiency reached an impressive 70.5%. Remarkably, this efficiency improved to 81.5% under acidic conditions (pH 5.0) at the same temperature. The incorporation of modified PNAs retained the dimensions and morphology of plain HumAfFt and confirmed the uptake of the PNA-loaded PAs-HumAfFt system in MEG01 cells, which overexpress ferritin receptor. Furthermore, the release and biological activity of PNAs were validated in cancer cells by the effective silencing of the GAPDH gene when compared to siRNA. These findings highlight the potential of the negatively charged PNA_10‑mer_ to retain gene-silencing capability despite chemical modifications and limited length. The involvement of polyamine linkers in the encapsulation process was confirmed, enabling precise modulation of molecular interactions and reinforcing the ***PA3.2-HumAfFt*** system as a versatile carrier for the efficient encapsulation and controlled release of therapeutic agents under physiological conditions.

## Supplementary Material



## Notes

MS characterization of PNAs; synthetic procedure for the **PA3.2** linker; DLS, ζ-potential, and native-PAGE measurements; and β-actin and siRNA sequences (PDF)
