# Supplementary material for: Evaluation of Peptide Nucleic Acid Encapsulation in Ferritin Nanocages for Gene Silencing Applications
Source: Biomacromolecules. 2025 Oct 24;26(11):8040–50. doi: 10.1021/acs.biomac.5c01489 (PMC12606638; doi:10.1021/acs.biomac.5c01489)
Supplement: Supplementary file 1 [file bm5c01489_si_001.pdf]

Supporting Information

Evaluation of Peptide Nucleic Acid Encapsulation in Ferritin Nanocages for Gene Silencing Applications

Andrea Patrizia Falanga,[1‡] Maria Vittoria Farina,[2‡] Gabriele Cianfoni,[3] Lorenzo Barolo,[2,4] Chiara Di Meo,[3] Giulia Elizabeth Borsatti, [2] Francesca Ghirga,[3] Bruno Botta,[3] Luca Pisano,[3] Nicola Borbone,[1] Stefano D'Errico,[1] Alessio Paone, [2] Giorgia Oliviero,[5] Deborah Quaglio,[3]* and Paola Baiocco[2]*

[1]Department of Pharmacy, University of Naples Federico II, Via Domenico Montesano 49, 80131 Naples, Italy

[2]Department of Biochemical Sciences "Alessandro Rossi Fanelli", Sapienza University of Rome, P.le A. Moro 5, 00185 Rome, Italy

[3]Department of Chemistry and Technology of Drugs, Sapienza-University of Rome, Rome, 00185, Italy

[4]Department of Physiology and Pharmacology, Sapienza University of Rome, 00185 Rome, Italy

[5]Department of Molecular Medicine and Medical Biotechnology, University of Naples Federico II, Via Sergio Pansini 5, 80131 Naples, Italy

*Corresponding authors

‡ These authors contributed equally to this work.

Table of contents

**1.**	**MS Characterization of PNAs**	**3**
**2.**	**Synthesis of PA3.2**	**12**
**3.**	**DLS Measurements**	**13**
**4.**	**ζ-potential Measurements**	**13**
**5.**	**Native-PAGE**	**15**
**6.**	**siRNA Sequences**	**15**

1. MS Characterization of PNAs

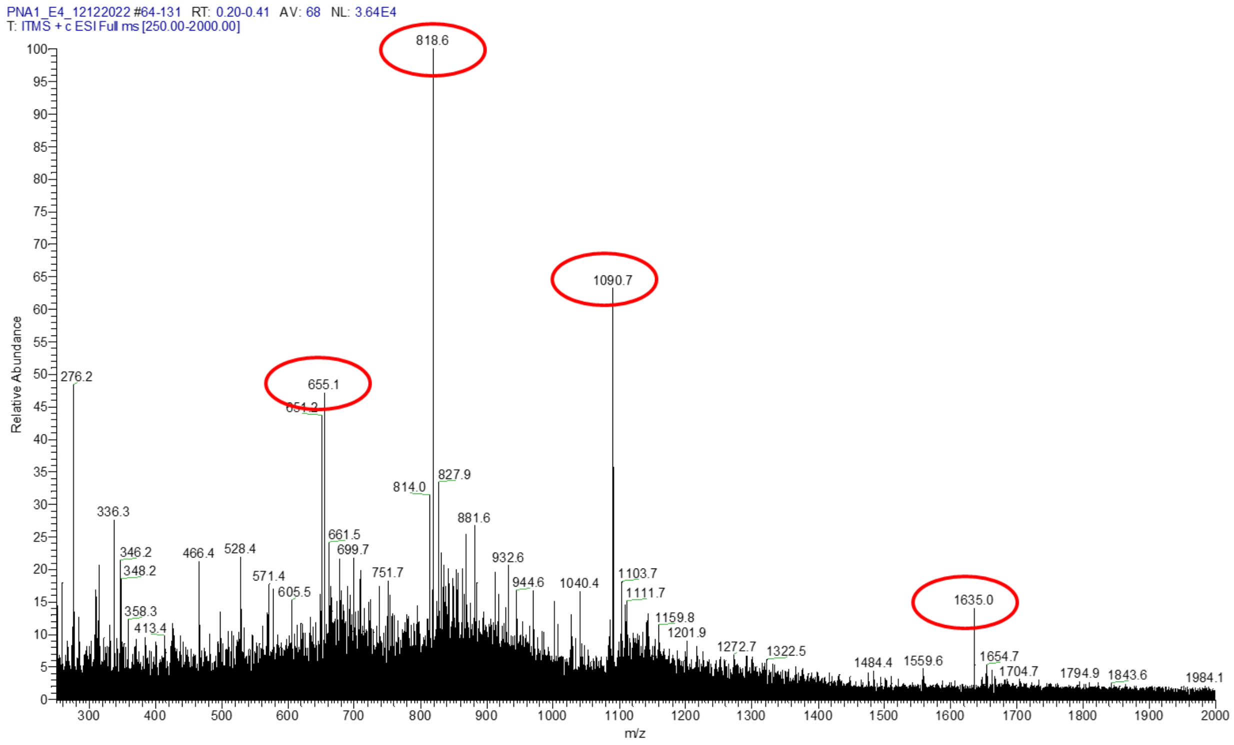

Figure S1: ESI-MS spectrum of **PNA$_{10\text{-mer}}$ E4 (–)** recorded in the positive ion mode. Calcd. for $[M + 2H]^{2+}$ 1634.6, found 1635.0; calcd. for $[M + 3H]^{3+}$ 1090.1, found 1090.7; calcd. for $[M + 4H]^{4+}$ 817.8, found 818.6, calcd. for $[M + 5H]^{5+}$ 654.5, found 655.1.

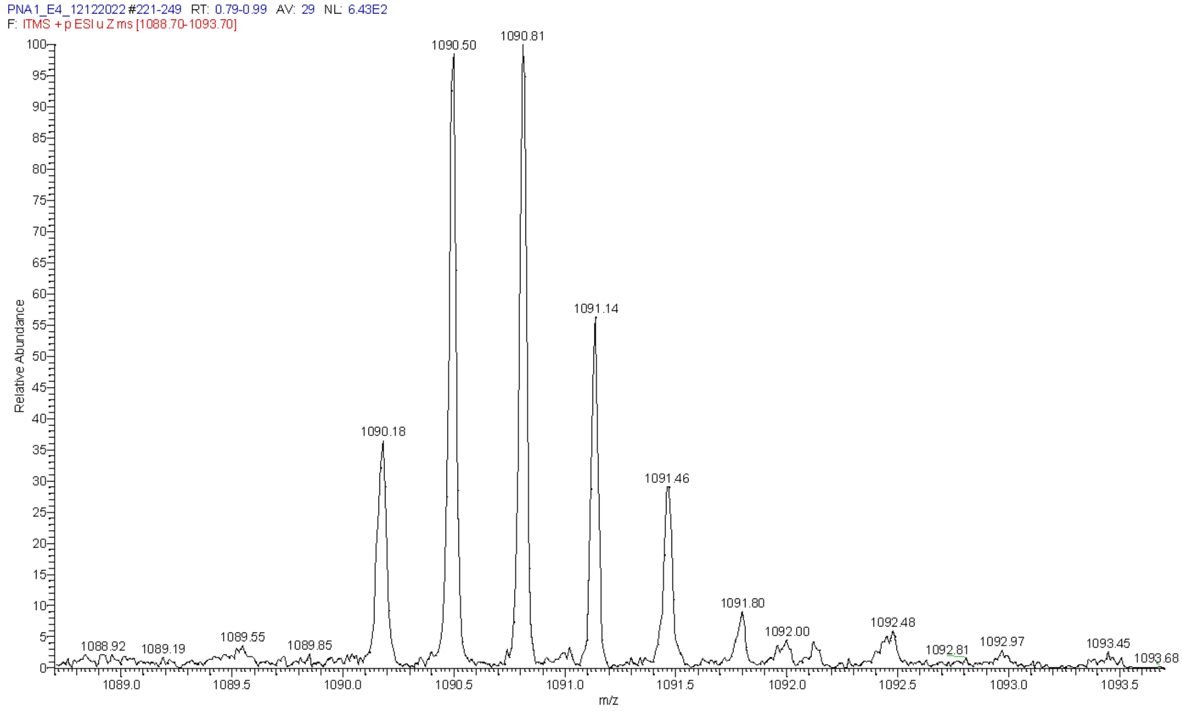

Figure S2: Expansion of the ESI-MS spectrum of **PNA$_{10\text{-mer}}$ E4 (–)** recorded in the positive ion mode. Calcd. for $[M + 2H]^{3+}$ 1090.1, found 1090.2.

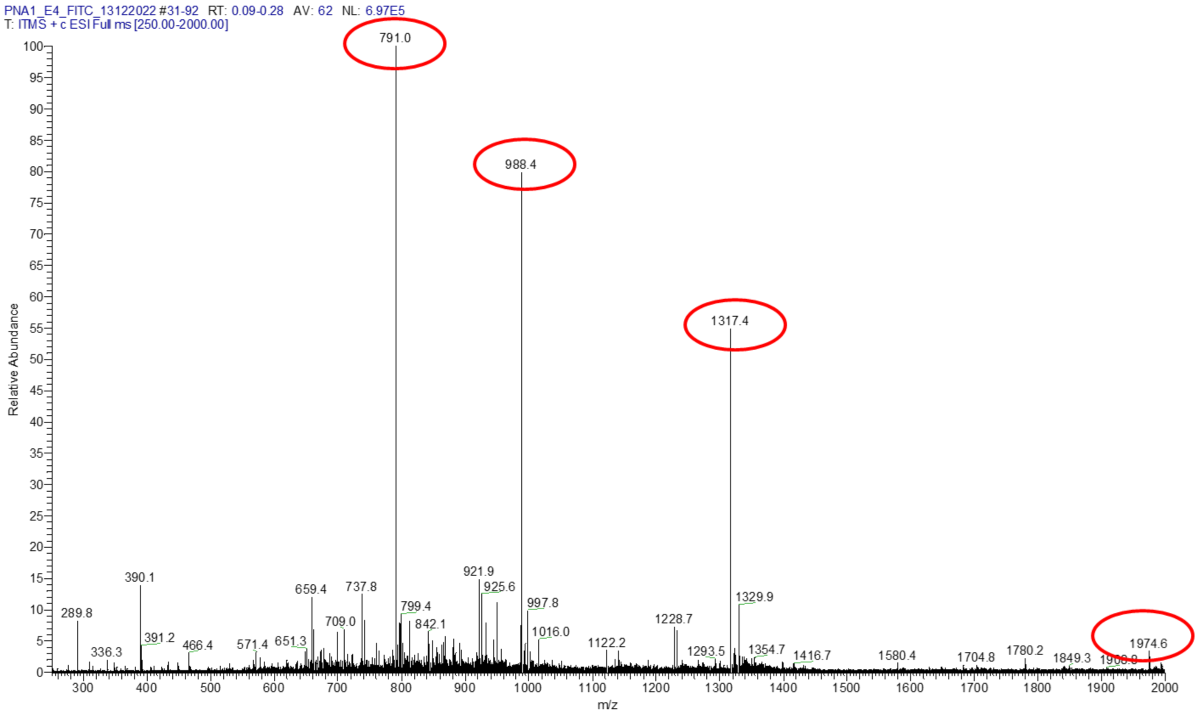

Figure S3: ESI-MS (m/z) of **FITC-PNA$_{10\text{-mer}}$ E4 (–)** recorded in the positive ion mode. Calcd. for $[M + 2H]^{2+}$ 1974.2, found 1974.6; calcd. for $[M + 3H]^{3+}$ 1317.5, found 1317.4; calcd. for $[M + 4H]^{4+}$ 987.6, found 988.4; calcd. for $[M + 5H]^{5+}$ 790.3, found 791.0.

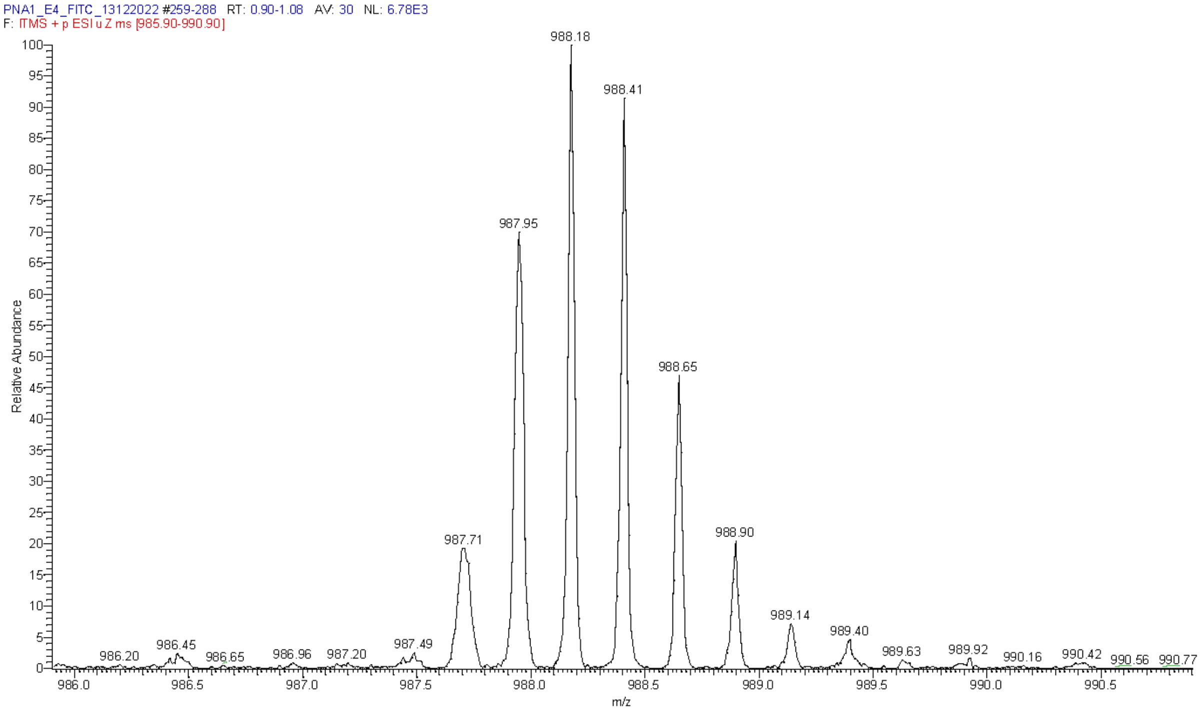

Figure S4: Expansion of the ESI-MS spectrum of **FITC-PNA$_{\mathbf{10\text{-}mer}}$ E4 (–)** recorded in the positive ion mode. Calcd. for $[M + 4H]^{4+}$ 987.6, found 987.7.

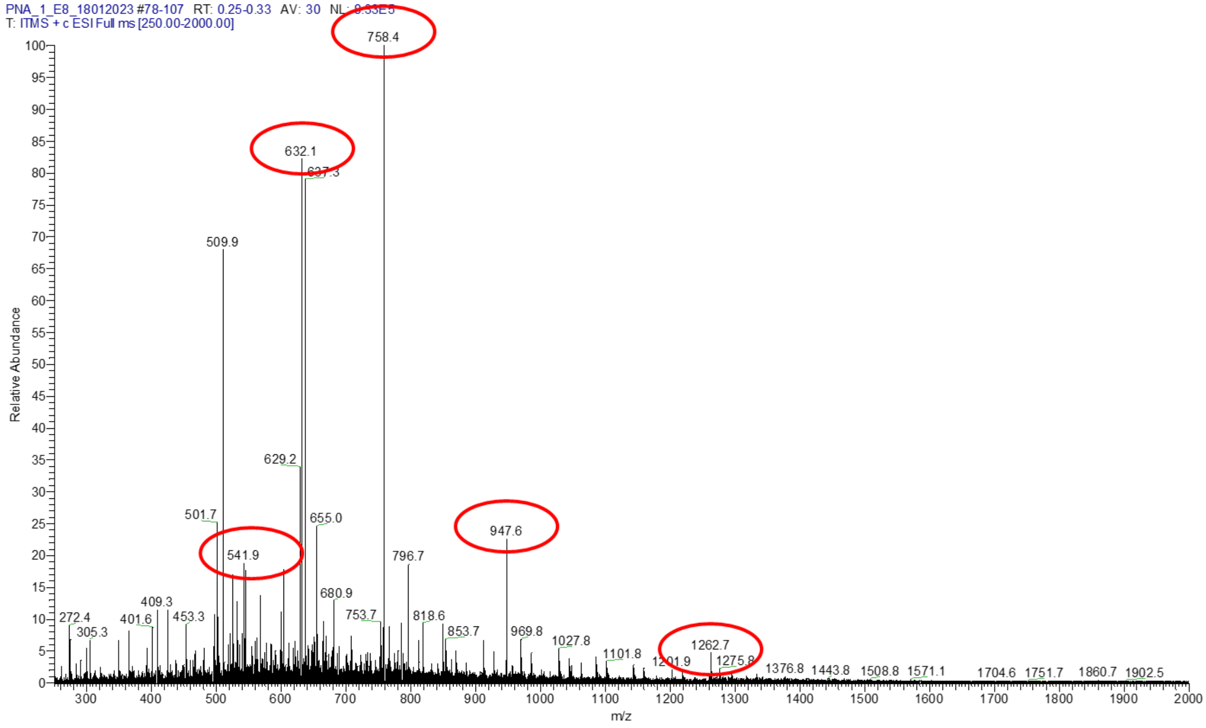

Figure S5: ESI-MS (m/z) of **PNA$_{\mathbf{10\text{-}mer}}$ E8 (–)** recorded in the positive ion mode. Calcd. for $[M + 3H]^{3+}$ 1262.2, found 1262.7; calcd. for $[M + 4H]^{4+}$ 946.9, found 947.6; calcd. for $[M + 5H]^{5+}$ 757.7, found 758.4; calcd. for $[M + 6H]^{6+}$ 631.6, found 632.1; calcd. for $[M + 7H]^{7+}$ 541.5, found 541.9.

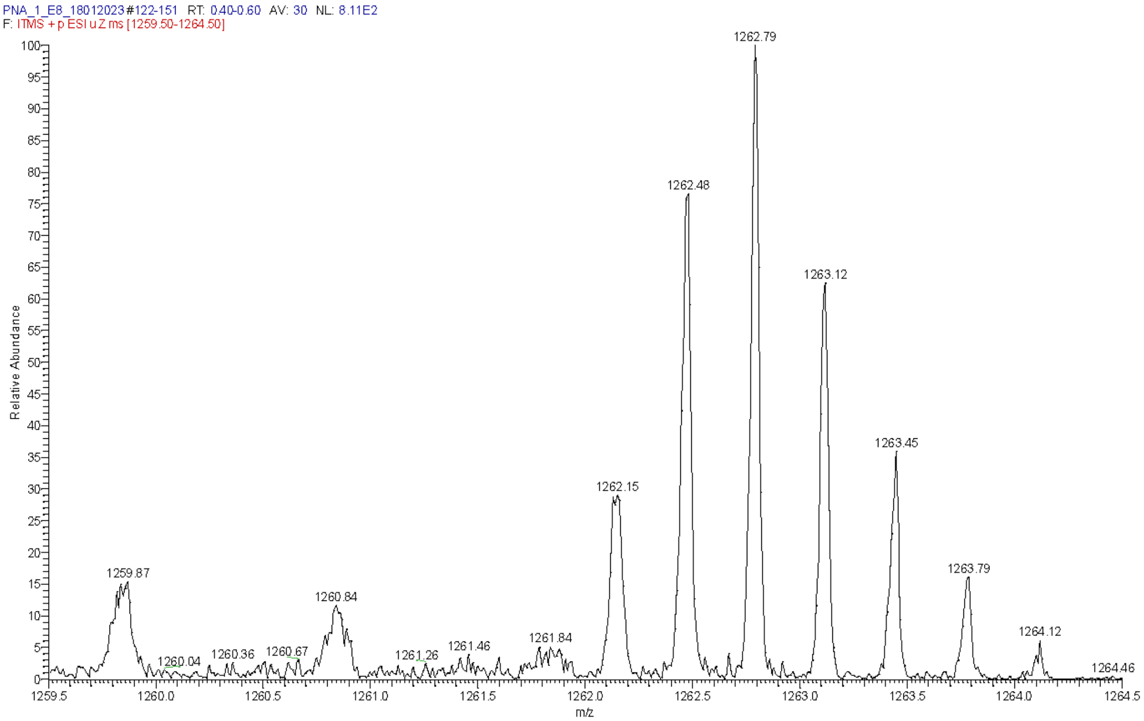

Figure S6: Expansion of the ESI-MS spectrum of **PNA$_{10\text{-mer}}$ E8 (–)** recorded in the positive ion mode. Calcd. for $[M + 3H]^{3+}$ 1262.2, found 1262.2.

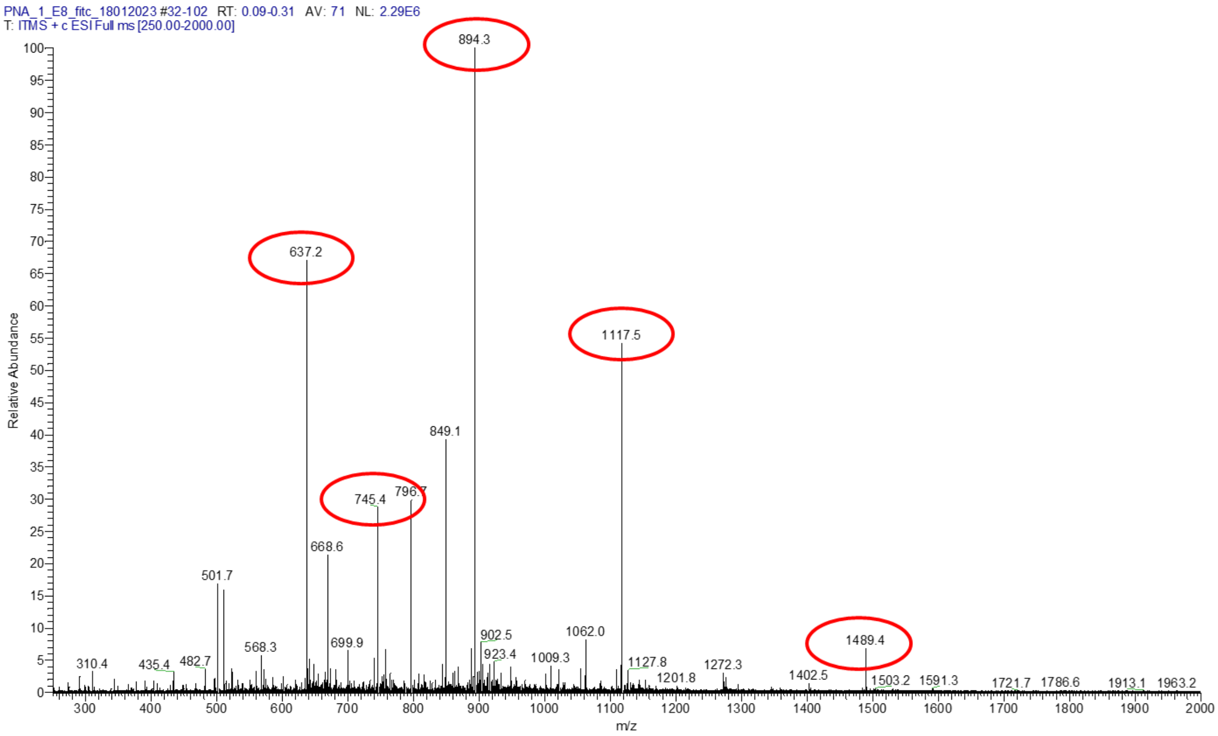

Figure S7: ESI-MS (m/z) of **FITC-PNA$_{10\text{-mer}}$ E8 (–)** recorded in the positive ion mode. Calcd. for $[M + 3H]^{3+}$ 1488.5, found 1489.4; calcd. for $[M + 4H]^{4+}$ 1116.7, found 1117.5; calcd. for $[M + 5H]^{5+}$ 893.5, found 894.3; calcd. for $[M + 6H]^{6+}$ 744.8, found 745.4; calcd. for $[M + 7H]^{7+}$ 638.5, found 637.2.

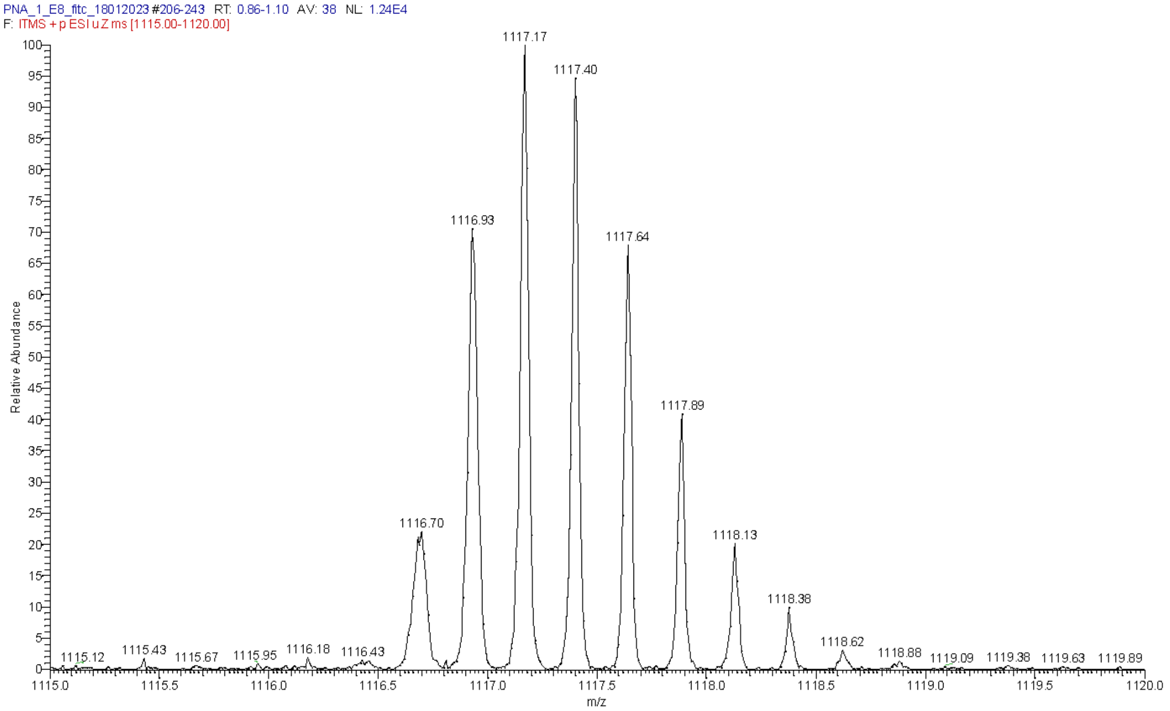

Figure S8: Expansion of the ESI-MS spectrum of **FITC-PNA$_{\mathbf{10\text{-}mer}}$ E8 (–)** recorded in the positive ion mode. Calcd. for $[M + 4H]^{4+}$ 1116.7, found 1116.7.

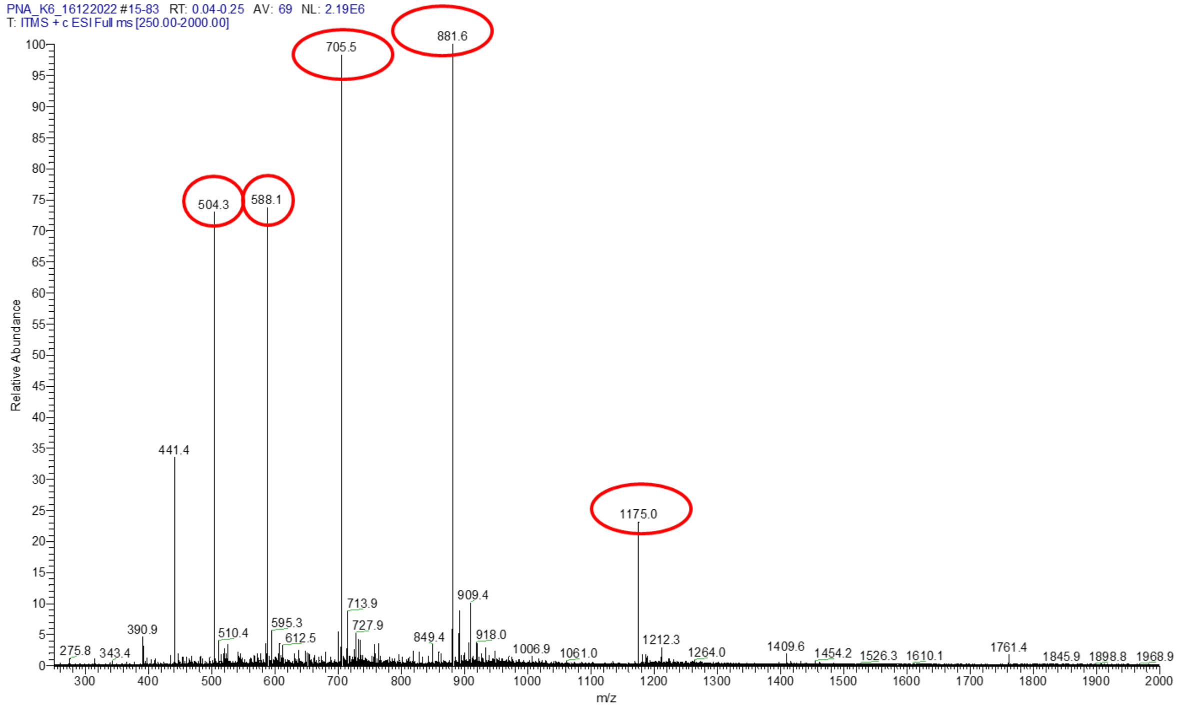

Figure S9: ESI-MS (m/z) of **PNA$_{\mathbf{10\text{-}mer}}$ K6 (+)** recorded in the positive ion mode. Calcd. for $[M + 3H]^{3+}$ 1174.2, found 1175.0; calcd. for $[M + 4H]^{4+}$ 880.9, found 881.6; calcd. for $[M + 5H]^{5+}$ 704.9, found 705.5; calcd. for $[M + 6H]^{6+}$ 587.6, found 588.1; calcd. for $[M + 7H]^{7+}$ 503.8, found 504.3.

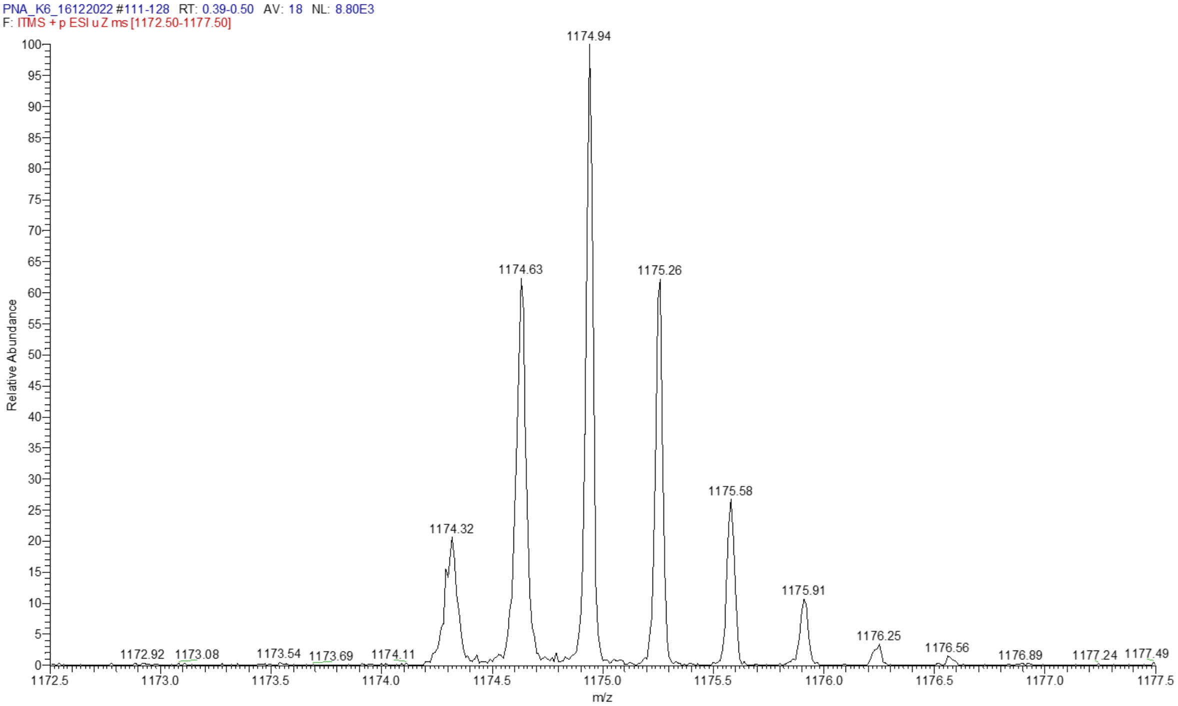

Figure S10: Expansion of the ESI-MS spectrum of **PNA$_{10\text{-mer}}$ K6 (+)** recorded in the positive ion mode. Calcd. for $[M + 3H]^{3+}$ 1174.2, found 1174.3.

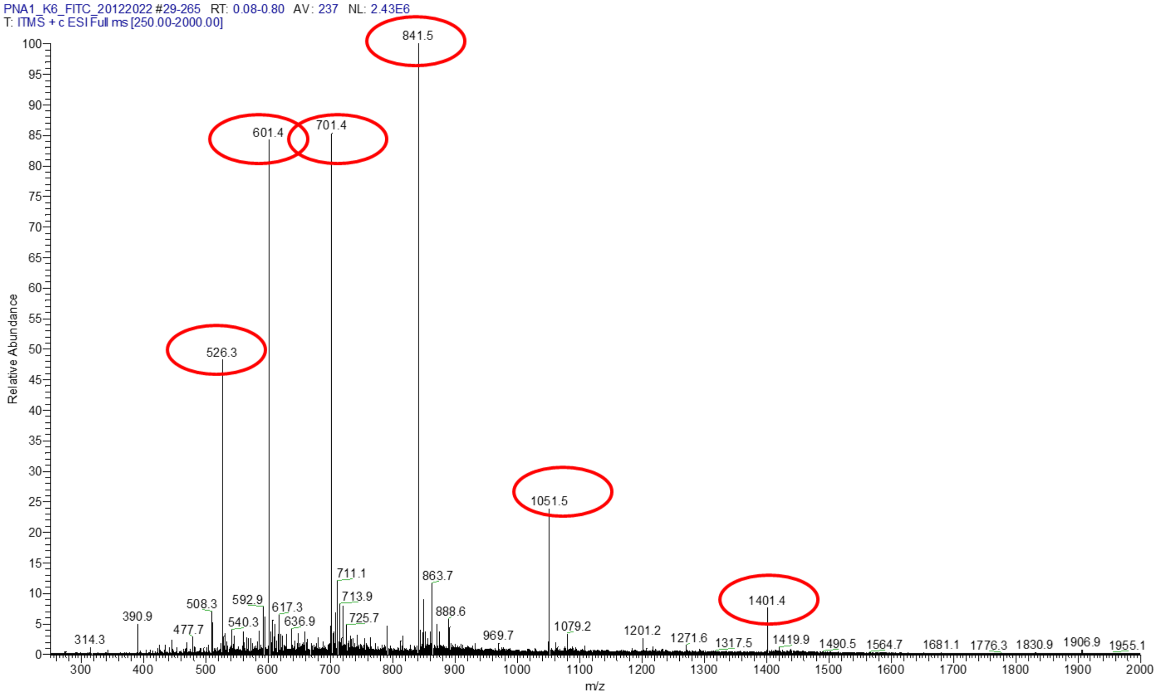

Figure S11: ESI-MS (m/z) of **FITC-PNA$_{10\text{-mer}}$ K6 (+)** recorded in the positive ion mode. Calcd. for $[M + 3H]^{3+}$ 1400.6, found 1401.4; calcd. for $[M + 4H]^{4+}$ 1050.7, found 1051.5; calcd. for $[M + 5H]^{5+}$ 840.8, found 841.5; calcd. for $[M + 6H]^{6+}$ 700.8, found 701.4; calcd. for $[M + 7H]^{7+}$ 600.8, found 601.4; calcd. for $[M + 8H]^{8+}$ 525.9, found 526.3.

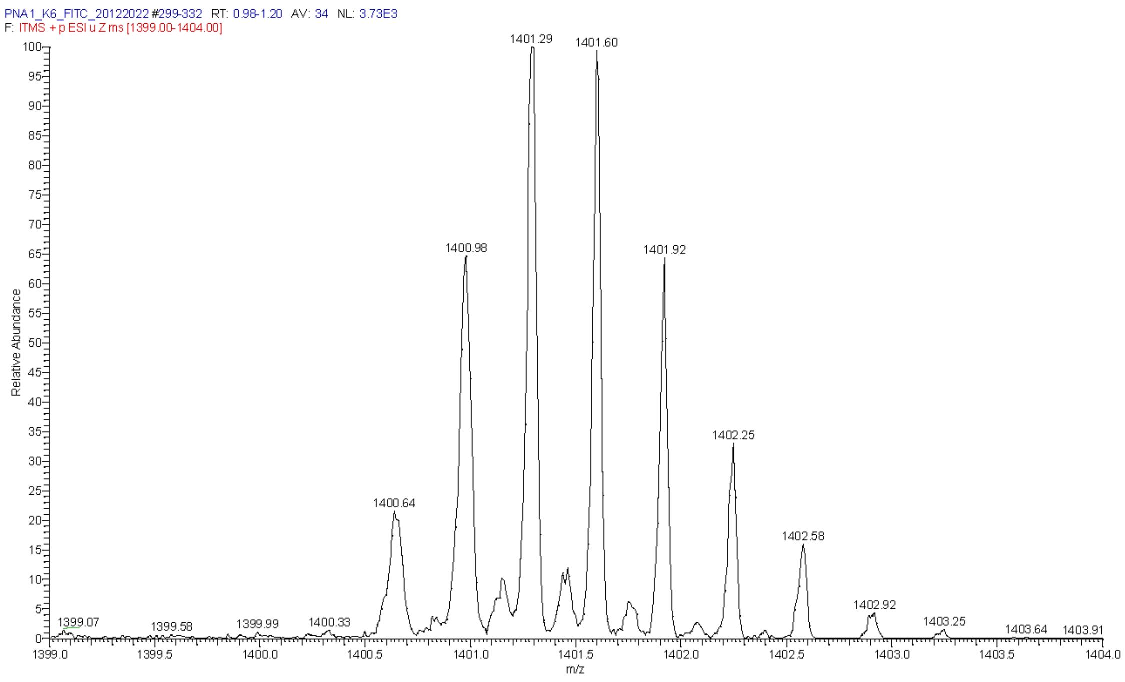

Figure S12: Expansion of the ESI-MS spectrum of **FITC-PNA$_{10\text{-mer}}$ K6 (+)** recorded in the positive ion mode. Calcd. for $[M + 3H]^{3+}$ 1400.6, found 1400.6.

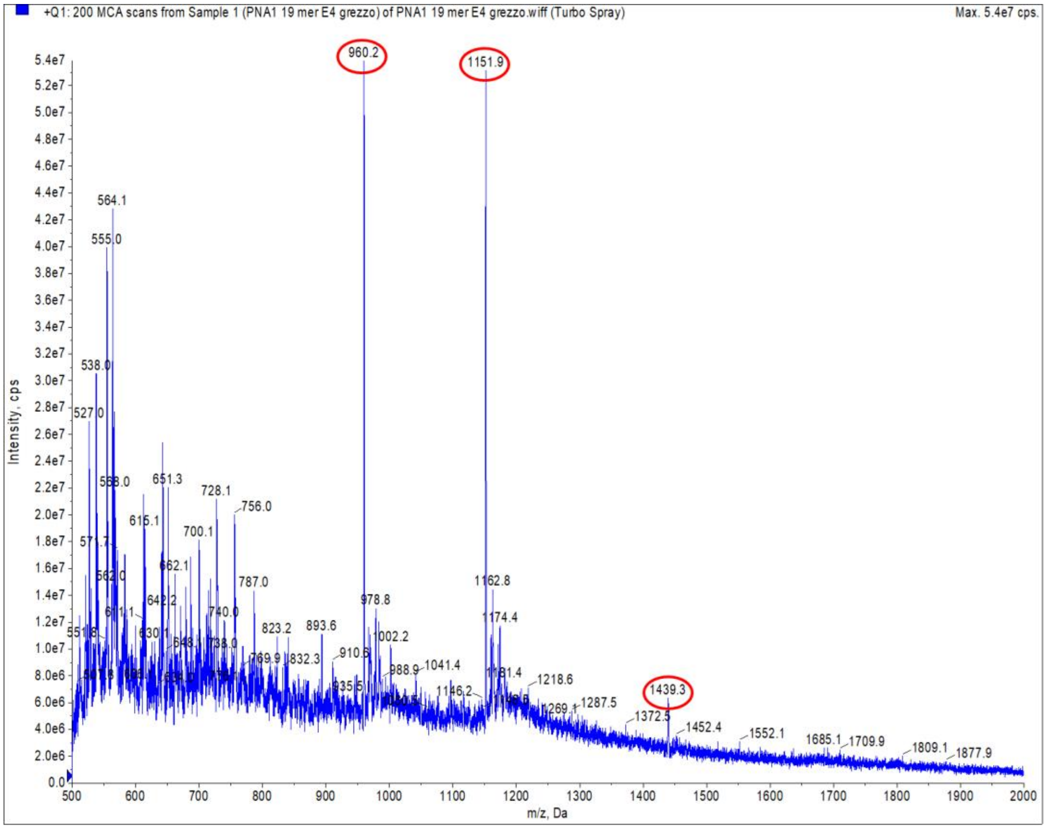

Figure S13: ESI-MS (m/z) of **PNA$_{19\text{-mer}}$ E4 (–)** recorded in the positive ion mode. Calcd. for $[M + 4H]^{4+}$ 1438.1, found 1439.3; calcd. for $[M + 5H]^{5+}$ 1050.7, found 1051.5; calcd. for $[M + 6H]^{6+}$ 959.0, found 960.2.

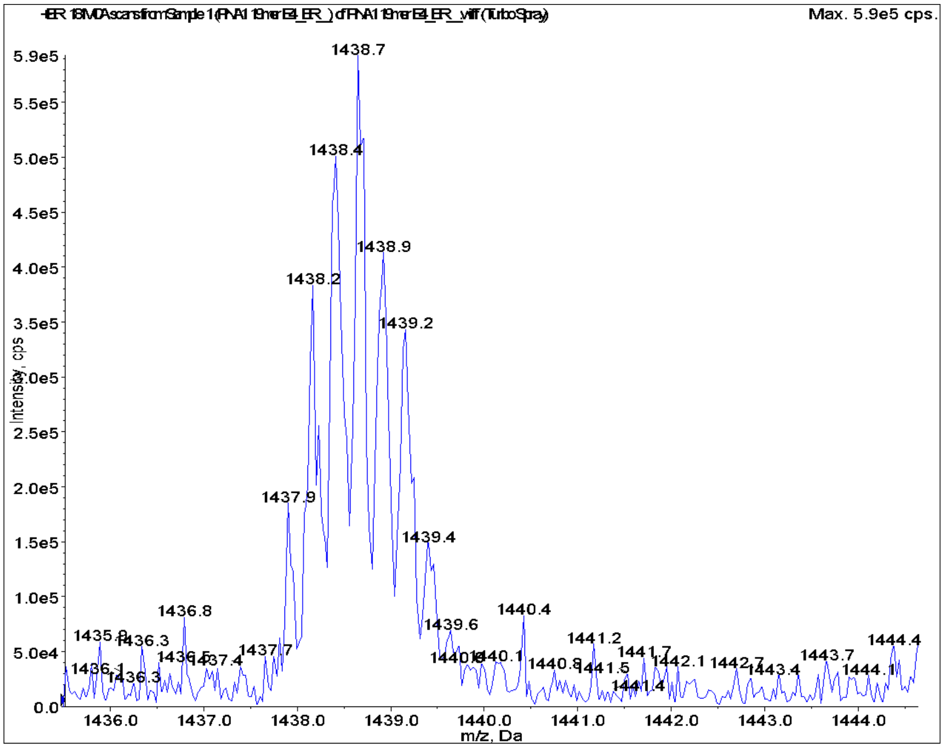

Figure S14: Expansion of the ESI-MS spectrum of **PNA$_{19\text{-mer}}$ E4 (–)** recorded in the positive ion mode. Calcd. for $[M + 4H]^{4+}$ 1438.1, found 1437.9.

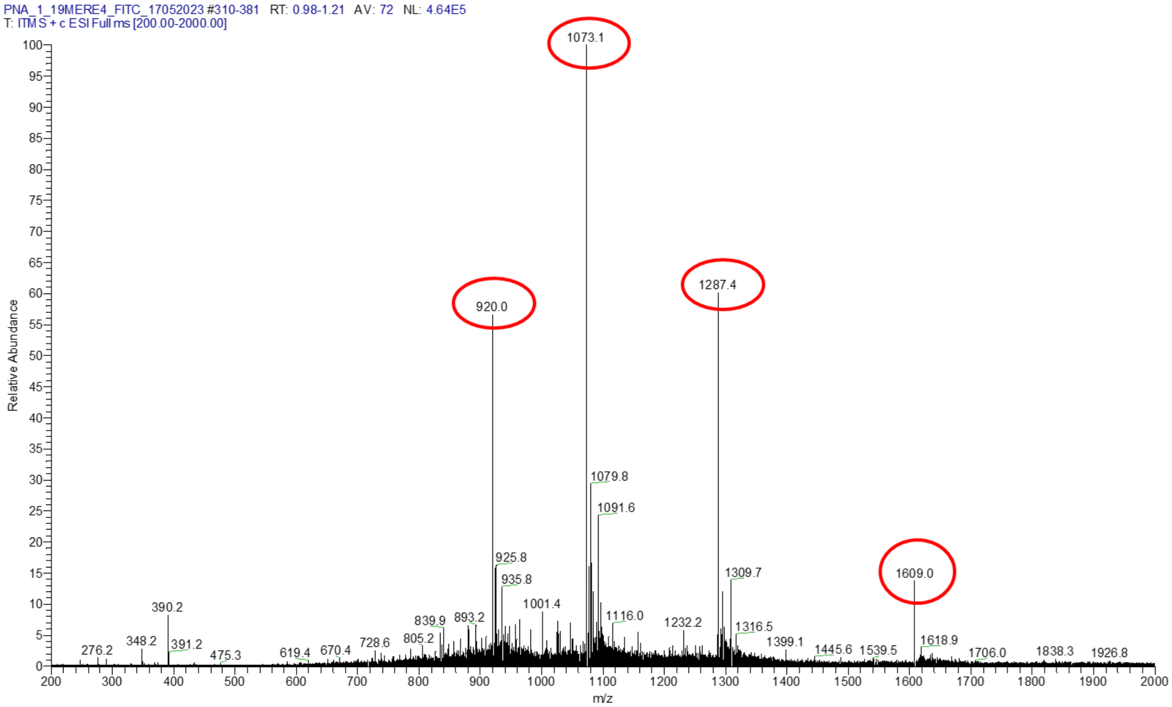

Figure S15: ESI-MS (m/z) of **FITC-PNA$_{19\text{-mer}}$ E4 (–)** recorded in the positive ion mode. Calcd. for $[M + 4H]^{4+}$ 1608.7, found 1609.0; calcd. for $[M + 5H]^{5+}$ 1286.5, found 1287.4; calcd. for $[M + 6H]^{6+}$ 1072.8, found 1073.1; calcd. for $[M + 7H]^{7+}$ 919.7, found 920.0.

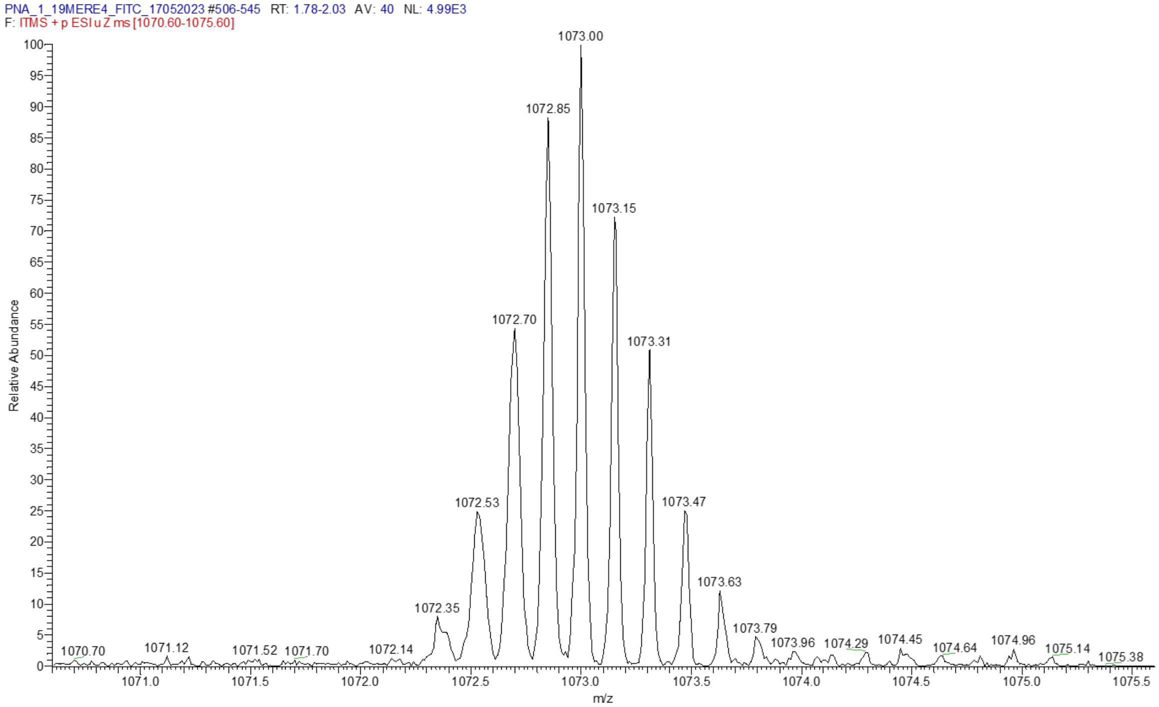

Figure S16: Expansion of the ESI-MS spectrum of **FITC-PNA$_{\mathbf{19\text{-}mer}}$ E4 (–)** recorded in the positive ion mode. Calcd. for $[M + 6H]^{6+}$ 1072.8, found 1072.4.

2. Synthesis of PA3.2

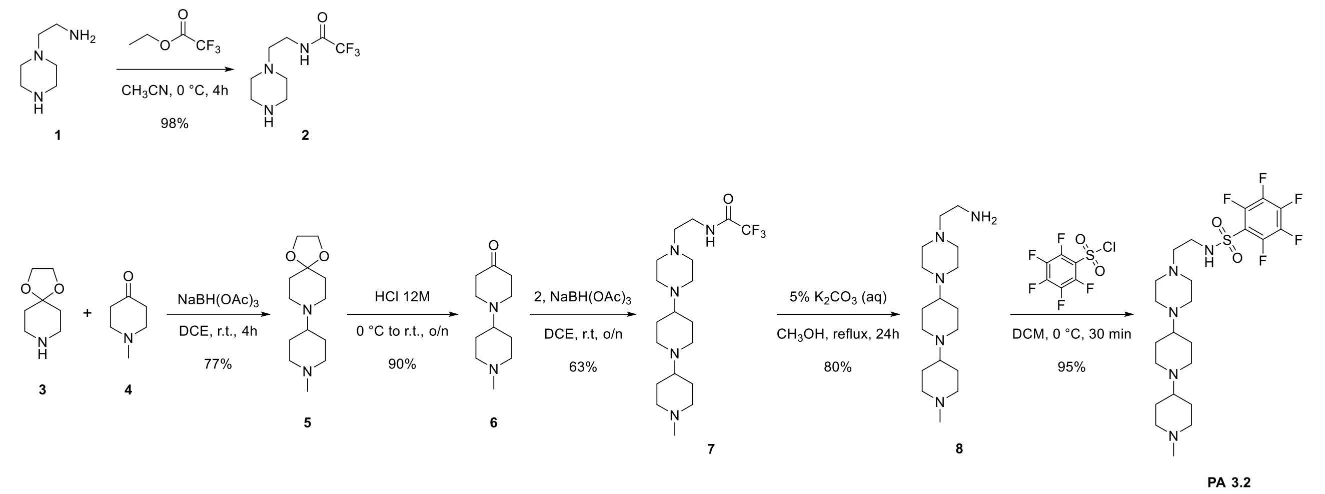

Scheme S1. Synthetic strategy for the obtainment of PA3.2.

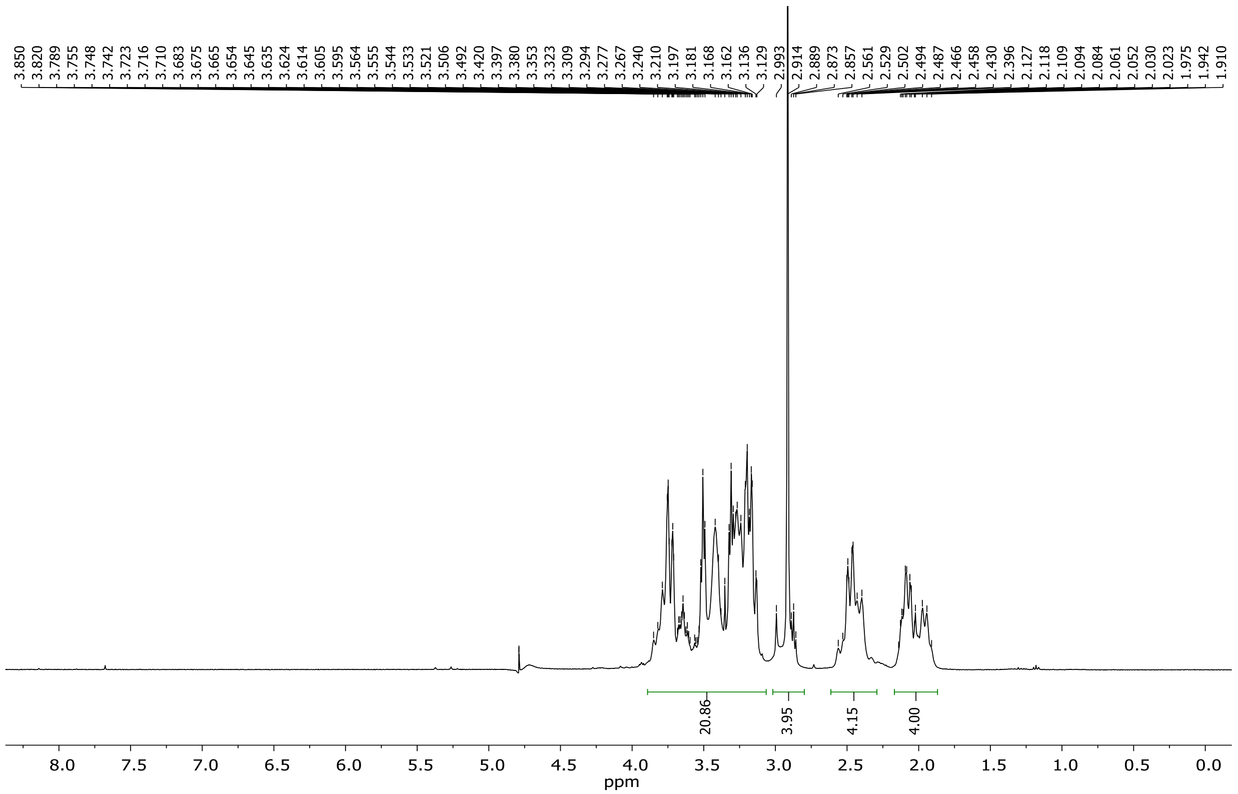

Figure. ^{1}H NMR of PA3.2 in D_2O.

^{1}H NMR (400 MHz, D_2O) δ 3.89 – 3.06 (m, 21H), 3.02 – 2.80 (m, 4H), 2.61 – 2.29 (m, 4H), 2.17 – 1.87 (m, 4H).

3. DLS Measurements

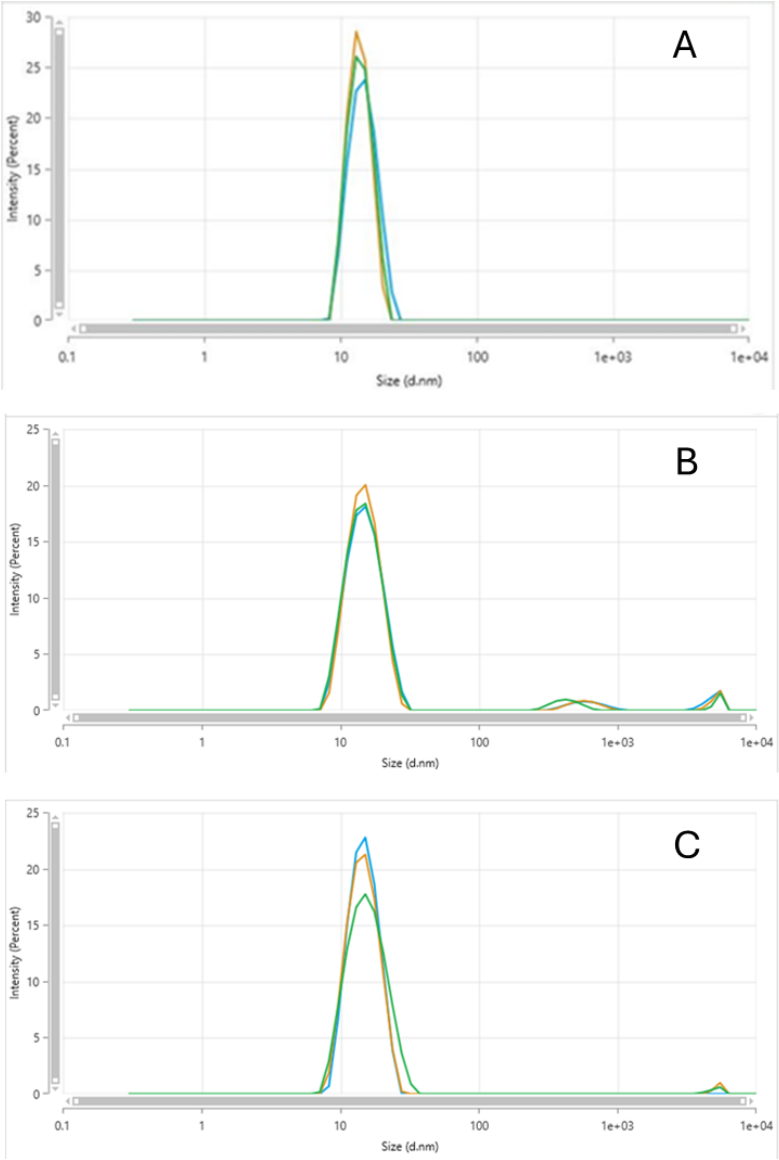

Figure S18: DLS profiles of HumAfFt (A), PA3.2-HumAfFt/$\mathbf{PNA_{10\text{-}mer}}$ **E4 (–)** complex (B) and PA3.2-HumAfFt/$\mathbf{PNA_{19\text{-}mer}}$ **E4 (–)** complex (C), at 1 mg /ml in 20 mM Hepes and 50 mM $MgCl_2$.

4. ζ-potential Measurements

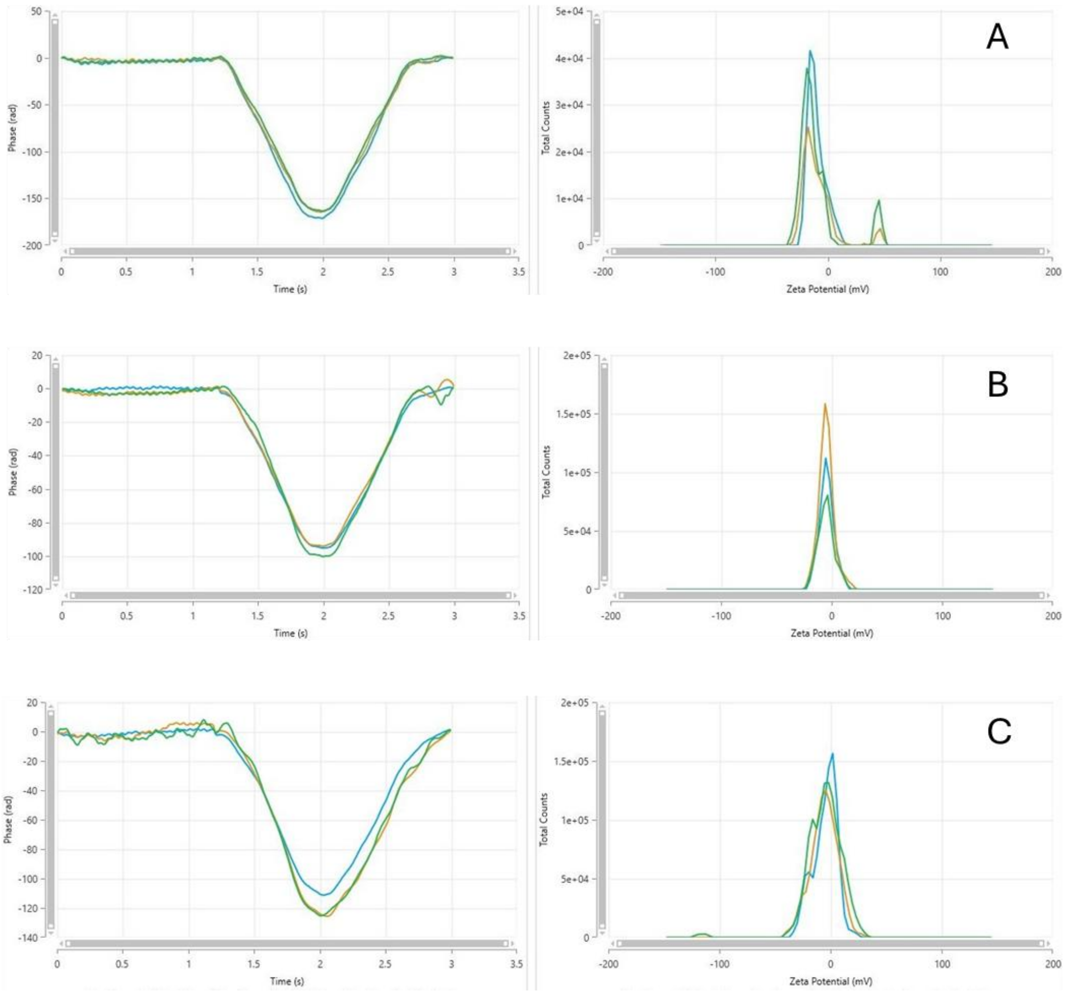

Figure S19: ζ-potential profiles of PA3.2-HumAfFt (A), **FITC-PNA$_{\mathbf{10\text{-}mer}}$ E4(–)**-PA3.2-HumAfFt at pH = 7.5 (B) **FITC-PNA$_{\mathbf{10\text{-}mer}}$ E4(–)**-PA3.2-HumAfFt at pH = 5 (C), at 1 mg /ml in 20 mM Hepes and 50 mM $MgCl_2$.

5. Native-PAGE

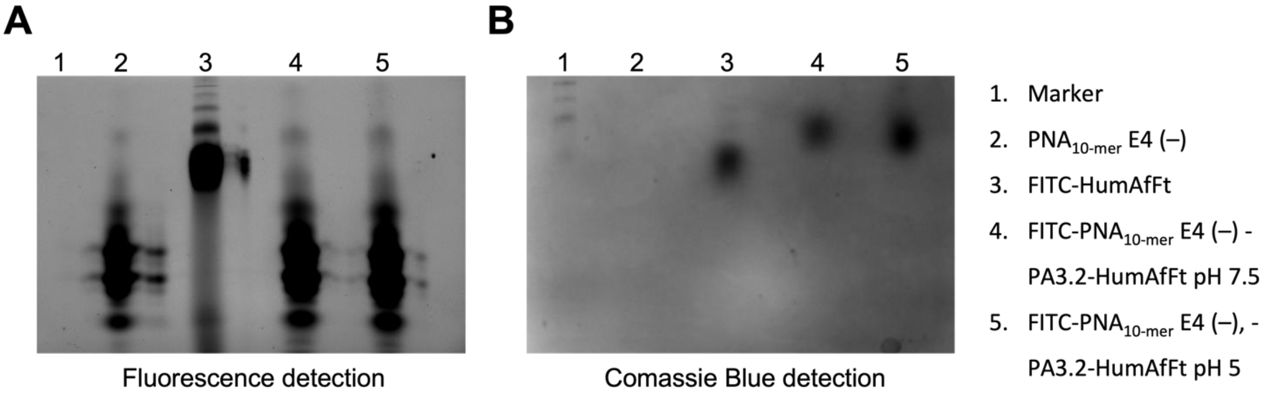

Figure S20: Native-PAGE analysis of FITC-PNA10-mer E4(–) encapsulation in PA3.2-HumAfFt, **(A)** Fluorescence detection of FITC-labeled samples **(B)** and Coomassie Blue staining for protein detection.

6. siRNA Sequences

siRNAs sequences targeting GAPDH were synthesized by Merck and purified by desalting chromatography.

siRNA Targeting GADPH	Sense sequence	Antisense sequence
	5'GGUUUACAUGUUCCAAUAU[dT][dT]	5'AUAUUGGAACAUGUAAACC[dT][dT]
	5'CUGACCUGCCGUCUAGAAA[dT][dT]	5'UUUCUAGACGGCAGGUCAG[dT][dT]
	5'GUCAACGGAUUUGGUCGUA[dT][dT]	5'UACGACCAAAUCCGUUGAC[dT][dT]
	5'CACAUGGCCUCCAAGGAGU[dT][dT]	5'ACUCCUUGGAGGCCAUGUG[dT][dT]
	Forward	Reverse
β-ACTIN	AATGTGGCCGAGGACTTTGAT	AGGATGGCAAGGGACTTCCTG
